# PAK2–c-Myc–PKM2 axis plays an essential role in head and neck oncogenesis via regulating Warburg effect

**DOI:** 10.1038/s41419-018-0887-0

**Published:** 2018-08-01

**Authors:** Amit Gupta, Athira Ajith, Smriti Singh, Rajendra Kumar Panday, Atul Samaiya, Sanjeev Shukla

**Affiliations:** 1 Epigenetics and RNA Processing Lab, Department of Biological Sciences, Indian Institute of Science Education and Research, Bhopal, Madhya Pradesh 462066 India; 2 Department of Radiotherapy, Bansal Hospital, Bhopal, Madhya Pradesh 462016 India; 3 Department of Surgical Oncology, Bansal Hospital, Bhopal, Madhya Pradesh 462016 India; 4 Present Address: Lab No. 315, Department of Biotechnology, Bhupat and Jyoti Mehta School of Biosciences, Indian Institute of Technology, Madras, Tamil Nadu 600036 India

## Abstract

The histone modifiers (HMs) are crucial for chromatin dynamics and gene expression; however, their dysregulated expression has been observed in various abnormalities including cancer. In this study, we have analyzed the expression of HMs in microarray profiles of head and neck cancer (HNC), wherein a highly significant overexpression of p21-activated kinase 2 (PAK2) was identified which was further validated in HNC patients. The elevated expression of PAK2 positively correlated with enhanced cell proliferation, aerobic glycolysis and chemoresistance and was associated with the poor clinical outcome of HNC patients. Further, dissection of molecular mechanism revealed an association of PAK2 with c-Myc and c-Myc-dependent PKM2 overexpression, wherein we showed that PAK2 upregulates c-Myc expression and c-Myc thereby binds to PKM promoter and induces PKM2 expression. We observed that PAK2–c-Myc–PKM2 axis is critical for oncogenic cellular proliferation. Depletion of PAK2 disturbs the axis and leads to downregulation of c-Myc and thereby PKM2 expression, which resulted in reduced aerobic glycolysis, proliferation and chemotherapeutic resistance of HNC cells. Moreover, the c-Myc complementation rescued PAK2 depletion effects and restored aerobic glycolysis, proliferation, migration and invasion in PAK2-depleted cells. The global transcriptome analysis of PAK2-depleted HNC cells revealed the downregulation of various genes involved in active cell proliferation, which indicates that PAK2 overexpression is critical for HNC progression. Together, these results suggest that the axis of PAK2–c-Myc–PKM2 is critical for HNC progression and could be a therapeutic target to reduce the cell proliferation and acquired chemoresistance and might enhance the efficacy of standard chemotherapy which will help in better management of HNC patients.

## Introduction

Head and neck cancer (HNC) is one of the most common and highly aggressive malignancy and the eighth most common cancer worldwide^1,2^. The global incidence of all HNCs has been estimated to be 4–6 × 10^5^ with the mortality rate of 2.2–3 × 10^5^ per year^3^. In Southeast Asian countries, notably India^4^, the occurrence of HNC is high among male population^5^ and is associated with late diagnosis as well as poor prognosis. With the advancement of surgical^6^ and radiation therapies^7^ the quality of HNC patient’s life has improved over the time. However, despite the improvement of health care systems the survival rate of HNC patients remains poor^8,9^, which highlights the need for new molecular targets for HNC treatment.

Epigenetic mechanisms play an important role in the cellular development and maintenance of cellular homeostasis. Any alteration of epigenetic mechanisms via the changes in DNA methylation^10^ and histone modification^11^ may lead to various diseases including cancer^12^. Various histone modifications are globally altered in different cancers, which promote cancer development^13^ and chemotherapeutic resistance^14^ and confer poor prognosis^15,16^. The cancer-associated changes in histone modifications might occur due to altered expression of histone modifiers (HMs)^17^ that may deregulate the gene regulation in favor of oncogenic growth. Accordingly, the perturbations of several HMs, such as class I histone deacetylases^18,19^, histone demethylases, KDM1A^9^ as well as histone methyltransferases EZH2^20^, are associated with cancer progression and confer poor prognosis. Therefore, to identify the deregulated HMs in HNC, we first enlisted all HMs using HIstome database^21^. Sequentially, the expression of all HMs was analyzed in HNC microarray profile available with Gene Expression Omnibus (GEO). For further studies, we selected upregulated HMs wherein we found a highly significant overexpression of p21-activated kinase 2 (PAK2). PAK2 is a member of PAK family of serine/threonine kinases, initially identified as a binding partner of the Rho GTPases, Cdc42 and RacI^22^. The PAK2 plays a critical role in many fundamental cellular functions, including chromatin remodeling, cytoskeletal remodeling, proliferation and regulation of cellular apoptosis^23–26^. Furthermore, PAK2 has also been shown to affect the histone modifications^26–28^ resulting in the alteration of gene expression. Moreover, PAK2 overexpression is observed in various human malignancies^29,30^, and has been proposed as an independent prognostic marker for gastric cancer^31^. Collectively, these findings suggest an important role of PAK2 in carcinogenesis. However, the role of PAK2 in HNC development and the underlying molecular mechanism remains to be established.

In this study, we have investigated the molecular mechanism of PAK2-mediated oncogenesis. Importantly, we showed that PAK2 is associated with higher proliferation, Warburg effect and chemotherapeutic resistance. The PAK2 depletion restricted the growth of cancer cells and decreased the chemotherapeutic resistance. Importantly, we report the role of β-catenin-mediated upregulation of c-Myc in PAK2-dependent HNC oncogenesis. Moreover, c-Myc then occupies the promoter region of *PKM* gene and upregulates the pyruvate kinase M2 (PKM2) expression, which then favors the aerobic glycolysis and HNC cell proliferation and thereby leads to PAK2–c-Myc–PKM2 axis-driven HNC progression. In summary, we have shown a novel regulatory role of PAK2 in HNC development and a potential framework for HNC cancer therapy by targeting PAK2–c-Myc–PKM2 axis.

## Materials and methods

### Microarray data analysis

Gene expression profiles utilized in this study were collected from the GEO^32,33^. Microarray platform-specific probes were mapped to gene symbols with appropriate annotation files. The expression values of genes with more than one probe were averaged using DNA Chip Analyzer (dChip) software and considered for the analysis. PAK2 gene expression values were extracted from normalized HNC tumor profiles. The significant difference in gene expression between normal and tumor was calculated using Student’s *t*-test (two-tailed). *P* value less than 0.05 was considered as significant. GraphPad Prism5 (La Jolla, CA, USA) was used to generate the boxplots.

### Survival curve

Overall survival information of the HNC patients was obtained from GEO (GSE42743) and was considered for predicting the association between PAK2 expression and patient survival. The patients were classified into low-expression and high-expression groups and Kaplan–Meier survival curve analysis was performed. The survival curve was plotted with GraphPad Prism5 (La Jolla, CA, USA).

### Cell culture

Human HNC cell lines H157 (ECACC 07030901), H413 (ECACC 06092007) and BICR10 (ECACC 04072103) were obtained from European Collection of Authenticated Cell Culture (ECACC) (Salisbury UK). All three cell lines were cultured in the ECACC recommended media, supplemented with 10% fetal bovine serum (Thermo Fisher Scientific, Waltham, MA, USA), 100 units/ml of penicillin and streptomycin (Thermo Fisher Scientific, Waltham, MA, USA), 2 mM l-glutamine (Sigma, Saint Louis, USA) and 0.5 µg/ml sodium hydrocortisone succinate. All three cell lines were cultured in a humidified atmosphere at 37 °C and 5% CO_2._

### Head and neck cancer sample collection

Informed consent was obtained from patients undergoing surgery for head and neck cancer at Bansal Hospital, Bhopal, India. After surgery, tumor and adjacent normal tissue pairs were collected and immediately snap frozen and stored at −80 °C until use. Tissues for RNA isolation were collected in RNA later (Thermo Fisher Scientific, Waltham, MA, USA) separately. This study was approved by the Institute Ethics Committee. Clinical characteristics of patients used in the study are presented in Supplementary Table S1.

### Oncomine data analysis

The expression of PAK2 was searched in Oncomine, and among various cancers, HNC profiles were selected for further investigation. The expression of PAK2 was analyzed in HNC normal and tumor tissue as well as in HNC cell lines as per experimental requirement. The analyzed expression data and graph were exported for representation.

### RNA interference

The H157, H413 and BICR10 HNC cells were infected with lentivirus containing small hairpin RNA (shRNA) purchased from Sigma (Saint Louis, USA) specific to PAK2 (shPAK2) and eGFP (shControl) with 8 µg/ml polybrene containing media. Cells were selected using 1 µg/ml puromycin for 3 days. Post selection cells were used for downstream experiments.

### Oligo sequence of shRNAs

**Table:** 

sheGFP	5′-CCGGTACAACAGCCACAACGTCTATCTCGAGATAGACGTTGTGGCTGTTGTATTTTT-3′
shPAK2	5′-CCGGCGGGATTTCTTAAATCGATGTCTCGAGACATCGATTTAAGAAATCCCGTTTTTTG-3′

### MKi67 staining

Post puromycin selection, the shControl and shPAK2 HNC cells (H157 and H413) were harvested and MKi67 staining was done using Alexa Fluor 488-labeled Ki67 antibody (ab197234, Abcam, Melbourne, Australia) as discussed previously^34^. The MKi67 flow cytometry experiment was done with fluorescence-activated cell sorting (FACS) Aria III by Becton Dickinson, (Franklin Lakes, NJ, USA) and expression as well as mean fluorescent intensity (MFI) were analyzed with FlowJo software version 10 (FlowJo, Ashland, OR, USA).

### Cell-cycle analysis

Post puromycin selection, the shControl and shPAK2 HNC cells (H157 and H413) were harvested and washed with 1× phosphate-buffered saline (PBS) twice. The cells were stained with propidium iodide (PI, BD Biosciences, India) in the presence of RNase and incubated in the dark for 30 min, as per manufacturer’s protocol. The samples were diluted with 1× PBS, and flow cytometry was performed using FACS Aria III by Becton Dickinson, (Franklin Lakes, NJ, USA) and data were analyzed using FlowJo software version 10 (FlowJo, Ashland, OR, USA).

### Quantitative reverse transcriptase-PCR (qRT-PCR)

Total RNA was extracted from cultured H157 cells, H413 cells and human HNC tumor and normal tissue samples using Trizol (Thermo Fisher Scientific, Waltham, MA, USA) according to the manufacturer’s instruction. RNA was quantified using Nanodrop (Thermo Fisher Scientific, Waltham, MA, USA). Then, 1 µg of total RNA was reverse transcribed by iScript complementary DNA (cDNA) synthesis kit (Bio-Rad, CA, USA) as per the manufacturer’s instructions. The experiment was done using SYBR green (Qiagen, Hilden, Germany) with light cycler 480 II (Roche, Basel, Switzerland) according to the manufacturer’s protocol. Primers were designed using the IDT Primer Quest tool (https://www.idtdna.com/). Primers used in this study are mentioned in the Supplementary Table S2. The average cycle thresholds of independent experiments were calculated and normalized to housekeeping control gene *RPS16* for HNC cells and *β*-*Actin* for HNC tissue samples using the formula: [2^^(Ct control – Ct target)^]. Student’s *t*-test was used to compare gene expression between two different groups. *P* < 0.05 was considered as statistically significant.

### Immunoblotting

Proteins were separated by sodium dodecyl sulfate–polyacrylamide gel electrophoresis and transferred to polyvinylidene difluoride (PVDF) membrane (Millipore). The protein containing PVDF membranes were probed with different primary antibodies: anti-PAK2 (2615, Cell Signaling Technology, Beverly, MA, USA), anti-c-Myc (9402, Cell Signaling Technology, Beverly, MA, USA), anti-CCND1 (ab134175, Abcam, Melbourne Australia), anti-PKM2 (4053, Cell Signaling Technology, Beverly, MA, USA), anti-Active β-Catenin (05665, Millipore, Burlington, USA), anti-GAPDH (5174, Cell Signaling Technology, Beverly, MA, USA), anti-β-Catenin (9562, Cell Signaling Technology, Beverly, MA, USA) and anti-flag (NBP1-06712SS, Novus Biologicals, Littleton, CO, USA) in a 1:1000 dilution. After 2 h of primary antibody incubation at room temperature, membranes were washed with 1× TBST (tris-buffered saline and Tween-20) and incubated with secondary antibodies Alexa-Flour 680 anti-rabbit IgG (A21109, Thermo Fisher Scientific, Waltham, MA, USA) and Alexa-Flour 790 goat anti-mouse IgG (A28182, Thermo Fisher Scientific, Waltham, MA, USA) for 30 min at room temperature. The membrane was washed, and bands were visualized using an Odyssey membrane Scanning system (Li-Cor Biosciences, Bad Homburg, Germany).

### Cell viability assay/MTT assay

Post puromycin selection, cells were seeded in 96-well culture plates (4 × 10^3^/well) for 12 h, 24 h, 36 h, 48 h and 60 h (in triplicate for each condition). The 20 µl MTT (Sigma, Saint Louis, USA) stock solution (2 mg/ml) was added to each well and incubated for 2–3 h. After the incubation, formazan crystals formed in the cells were solubilized using dimethyl sulfoxide and the optical density was analyzed at 600 and 750 nm using plate reader BioTek Eon (BioTek, Winooski, USA).

### Wound healing assay

Post puromycin selection, 1 × 10^5^ cells/well were seeded in 12-well plate and upon reaching at 100% confluency, wound was created using 200 μl pipette tip and washed with 1× PBS for two times to remove cellular debris. Wounds were visualized at 10× with an inverted microscope (Olympus, Tokyo, Japan) and three random images were captured for few days as indicated. Wound width was measured using Q-Capture software and graph was plotted with GraphPad Prism5 (La Jolla, CA, USA).

### Invasion assay

The puromycin selected 2 × 10^4^ cells were added to the upper chamber of transwell (Corning, NY, USA) over Matrigel (Corning, Bedford, MA, USA) layer and incubated for 24 h in cell culture incubator. The non-migrated cells in upper layer of Matrigel were removed and cells migrated to lower chamber of transwell were fixed in 4% paraformaldehyde, stained with 0.05% crystal violet and five random fields were counted using an inverted microscope (Olympus, Tokyo, Japan).

### Colony-forming assay

Post puromycin selection, cells were trypsinized, and 1 × 10^3^ cells were seeded in the new 6-well cell culture plate and maintained in 0.5 µg/ml puromycin containing media for 7 days. Cell colonies were visualized by crystal violet staining. For staining, cells were fixed using methanol and acetic acid (3:1) for 5 min and washed with 1× PBS three times. Cells were then stained with 0.05% crystal violet for 30 min. Post staining, cells were washed twice with 1× PBS and plates were dried for 30 min at room temperature and scanned with Epson Scanner. Colonies containing more than 50 individual cells were counted using microscope as well as relative intensity of each well and colony area was quantified using ImageJ software^35^ (La Jolla, CA, USA).

### Caspase 3/7 assay

Post puromycin selection, the 4 × 10^4^ cells/well were seeded in white color 96-well plate. Cells were treated with 5 μM camptothecin and 50 μM etoposide after 12 h of incubation. After 5 h of camptothecin treatment and 24 h of etoposide treatment, the caspase 3/7 activation was measured using the Caspase-Glo 3/7 Assay (Promega, Madison, USA) as recommended by the manufacturer. Luminescence readings were taken using Glomax multi-detection system (Promega).

### Annexin–PI staining

The cellular apoptosis in PAK2-depleted and control cells were analyzed using Annexin–FITC and PI staining kit (BD Biosciences, India) as per the manufacturer’s protocol. The flow cytometric analysis was done using FACS Aria III (Becton Dickinson), and data were analyzed using FlowJo software version 10 (FlowJo, Ashland, OR, USA).

### Lactate assay

Post puromycin selection, 3 × 10^5^ cells/well were seeded in 6-well plate. Cells were homogenized with lactate assay buffer after 48 h of seeding. Lactate quantification was performed using a commercially available lactate assay kit (Sigma, Saint Louis, USA) in a 96-well plate as per the manufacturer’s instruction. Lactate production was measured with plate reader (BioTek Eon) at an optical density of 570 nm and lactate was quantified as per the manufacturer’s protocol.

### Glucose uptake assay

Post puromycin selection, 3 × 10^5^ cells/well were seeded in 6-well plate. Cells were homogenized with glucose assay buffer after 48 h of seeding. Glucose level quantification was performed using a commercially available glucose assay kit (Abcam, Melbourne, Australia) in a 96-well plate as per the manufacturer’s protocol. Glucose uptake was measured with a plate reader (BioTek Eon) at an optical density of 570 nm.

### c-Myc and PAK2 overexpression construct generation

The overexpression plasmids were constructed by amplifying *c-Myc* and *PAK2* fragment from H157 cDNA using Platinum Q5 Hotstart High-Fidelity DNA Polymerase (New England Biolabs, MA, USA) using the primers mentioned in the Supplementary Table S2. The *c-Myc* product was cloned between the *Bam*HI and *Eco*RI sites of pAIP lentivirus system-based expression Vector (Addgene, MA, USA) and the *PAK2* was cloned between *Bam*HI and *Xho*I sites in pCMV-3Tag 1A plasmid (Agilent Technologies, Santa Clara, CA, USA).

### Chromatin immunoprecipitation (ChIP)

ChIP assays were performed as described previously^36^. Briefly, after puromycin selection, cells were sonicated, and chromatin (25 µg) was immunoprecipitated by adding the antibody of interest followed by overnight incubation at 4 °C. The following antibodies were used for ChIP: Anti-c-Myc and Normal Rabbit IgG (12–370, Millipore, Burlington, USA). Immunoprecipitated fractions and 5% input were analyzed by quantitative real-time PCR in duplicate using the SYBR Green Master Mix (Qiagen, Hilden, Germany) with specific primers (mentioned in the Supplementary Table S2) of predicted c-Myc binding regions. Normalization was performed to input using the formula: [2^^(Ct input – CT immunoprecipitation)^]. Resultant values were further normalized relative to the rabbit Ig control ChIP values for the primer set. Student’s *t*-test was used to identify the significance between two different groups*. P* value of <0.05 was considered statistically significant.

### ChIP-sequencing (ChIP-seq) data analysis

The ChIP-seq data were analyzed using the University of California Santa Cruz (UCSC) genome browser. The bigwig file of c-Myc ChIP-seq data was added in the custom track window provided in the UCSC genome browser, and the enrichment peak of c-Myc was analyzed at *PKM* gene promoter region.

### Immunocytochemistry (ICC)

Post puromycin selection, the ICC was performed by following the methodology provided by Abcam (http://www.abcam.com/protocols/immunocytochemistry-immunofluorescence-protocol). The expression of PKM2 which is directly proportional to GFP intensity was observed, and the images were captured at 10× using Evos FL Auto2 microscope (Thermo Fisher Scientific, Waltham, MA, USA). The intensity of the image was analyzed using ImageJ software (La Jolla, CA, USA).

### Human Transcriptome Array (HTA) 2.0 data analysis

The raw HTA 2.0 files were normalized and analyzed using Transcription Array Console. The expression index (linear) ≤ −2 or expression index (linear) ≥ + 2 with *P* < 0.05 were set as the criteria to identify the differentially expressed genes. The heat map was prepared through Morpheus, an online tool provided by Broad Institute (https://software.broadinstitute.org/morpheus/).

### Densitometric analysis

Densitometric analysis was performed using ImageJ software suit. Briefly, the band intensity was calculated with ImageJ and normalized with respective loading control. The normalized control values were further normalized to one, and the protein fold change was calculated using control values.

### Statistical analysis

The statistical analysis was performed with GraphPad Prism5 (La Jolla, CA, USA). In the bar graph, differences between two groups were compared using an unpaired two-tailed Student’s *t*-test. The differences between three or more groups were calculated using one-way analysis of variance by the Newman–Keuls test. The differences were considered statistically significant with *P < 0.05, **P < 0.01 and ***P < 0.001, ns non-significant difference (*P* > 0.05).

## Results

### PAK2 is overexpressed in HNC and confers poor clinical outcome

**Fig. 1:**
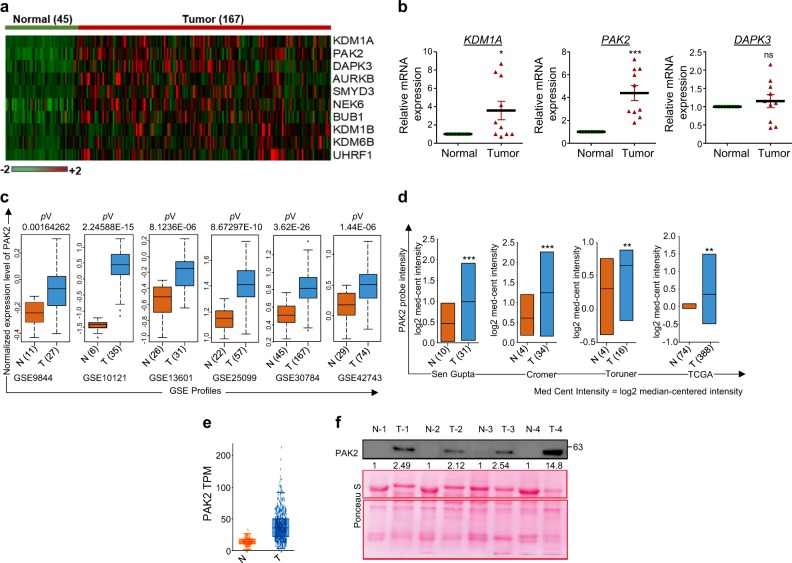
Detection and validation of PAK2 overexpression in head and neck tumor samples. **a** Heat map of top 10 overexpressed histone modifiers in head and neck normal and tumor samples (generated using DNA Chip analyzer). **b** Quantitative mRNA expression analysis of *KDM1A*, *PAK2* and *DAPK3* normalized to β-actin in10 normal and tumor samples of HNC patients. **c** Expression pattern of *PAK2* gene in independent cohorts of head and neck cancer gene expression profiles. Box plot shows the elevated expression of *PAK2* in head and neck tumor tissues compared to normal tissue. **d** Analysis of PAK2 expression in four different microarray profiles using Oncomine database. The *y*-axis represents log2-median centered intensity. **e** Analysis of PAK2 transcript per million (TPM) in normal and head and neck tumor samples through MiPanda (http://www.mipanda.org) cancer RNA-seq database. **f** Analysis of PAK2 protein expression in 26 normal and tumor tissue samples of HNC patients by PAK2 immunoblotting (also see Supplementary Figure S1g). N normal, T tumor, GSE Genomic Spatial Event. Error bars shows mean values ± SD. Differences were considered statistically significant with *P < 0.05, **P < 0.01 and ***P < 0.001, ns non-significant difference (*P* > 0.05)

In order to identify the overexpressed HMs in HNC, we first enlisted the HMs using HIstome database^21^. The expression of HMs was analyzed in HNC microarray profile (GSE30784). Significantly overexpressed HMs were shortlisted by the method shown in Supplementary Figure S1a. The expression of top 10 overexpressed HMs was represented as a heat map (Fig. 1a). Furthermore, top three overexpressed HMs, KDM1A, PAK2 and DAPK3, were selected, and their expression was cross-validated in ten HNC patient samples by qRT-PCR analysis, which showed a significant overexpression of PAK2 in comparison to KDM1A and DAPK3 (Fig. 1b) and was selected for further analysis. To strengthen our preliminary observation, the expression of PAK2 was further validated in five additional HNC microarray profiles, which revealed a highly significant upregulation of *PAK2* in HNC (Fig. 1c). Further validation using Oncomine database^37^ in three independent HNC studies^38–40^, The Cancer Genome Atlas (TCGA) database as well as RNA-seq data from MiPanda (Michigan Portal for the Analysis of NGS Data) database revealed a consistent overexpression of *PAK2* transcript (Fig. 1d, e and Supplementary Figure S1b) in HNC. Interestingly, analysis of TCGA data showed a positive correlation of *PAK2* expression with higher stages and grades of HNC (Supplementary Figure S1c,d). Additionally, the *PAK2* expression positively correlates with the higher T-staging (Supplementary Figure S1e,f), suggesting that the PAK2 could be responsible for the advancement of cancer.

To validate the results of in silico studies, we performed PAK2 immunoblotting in 26 HNC tumor samples, wherein 24 tumor samples showed PAK2 overexpression in comparison to normal counterpart (Fig. 1f, Supplementary Figure S1g). Additionally, Kaplan–Meier survival analysis showed that high PAK2 expressing HNC patients (GSE42743) had shorter overall survival than those with low expression, highlighting the prognostic value of PAK2 in HNC (Supplementary Figure S1h). Conclusively, these analyses strongly suggest a definite role of PAK2 overexpression in HNC progression.

### PAK2 deficiency leads to reduced proliferation of head and neck cancer cells

**Fig. 2:**
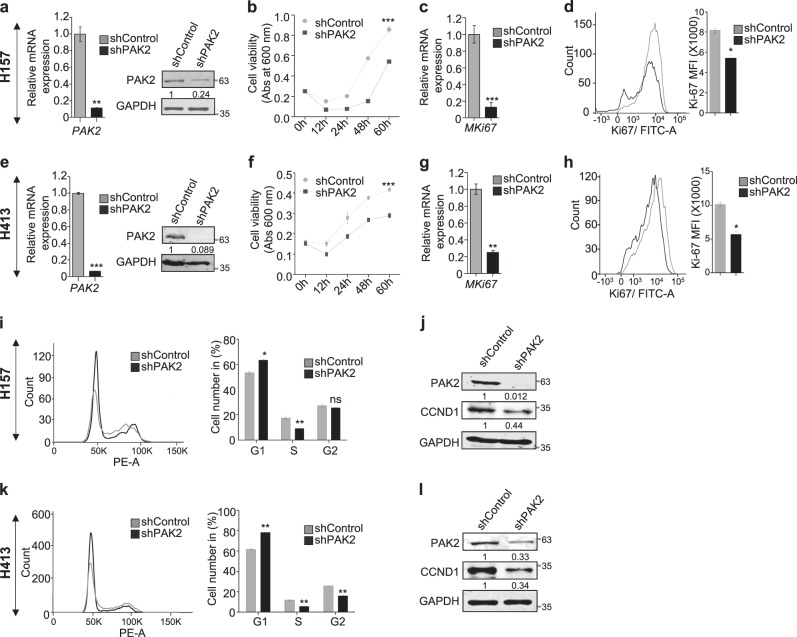
PAK2 affects proliferation of head and neck cancer cells. PAK2 expression was depleted in two types of HNC cell lines (H157 and H413), and differential proliferation status of these cell lines was analyzed. **a**, **e** Knockdown of PAK2 expression was analyzed at mRNA and protein levels (*n* = 3). **b**, **f** Relative cell proliferation was analyzed through MTT assay (*n* = 3). **c**, **g** mRNA and **d**, **h** protein expression status of cell proliferation marker Ki67 (left) was analyzed through qPCR and flow cytometer, respectively. (Right) Mean fluorescence intensity (MFI) of Alexa Fluor 488-Ki67 was measured using software FlowJo 10 (*n* = 3). **i**, **k** (Left) Cell-cycle stages were analyzed using flow cytometer (representative image of 3 independent experiment) (right) bar graph shows average of three independent experiments and **j**, **l** cyclin D1 (CCND1) protein expression level was analyzed through immunoblotting (*n* = 3). Abs absorbance. Samples in (**a**–**c**) and (**e**–**g**) were analyzed in duplicate. Error bars shows mean values ± SD. Differences were considered statistically significant with *P < 0.05, **P < 0.01 and ***P < 0.001, ns non-significant difference (*P* > 0.05)

Upon observing an elevated expression of PAK2 in HNC, first we analyzed the expression of PAK2 in three HNC cells and observed the comparable expression of PAK2 in H413 and H157 cells, but relatively less expression in BICR10 cells (Supplementary Figure S2a). Next, we examined the role of PAK2 in HNC cell proliferation by depleting PAK2 in three HNC cancer cell lines with shRNA against PAK2 (shPAK2) or against eGFP (shControl) (Fig. 2a, e, and Supplementary Figure S2b) and observed significantly reduced viability in PAK2-depleted cells (Fig. 2b, f, and Supplementary Figure S2c). Furthermore, we also observed reduced cell proliferation of HNC cells upon PAK2 depletion by cell counting assay (Supplementary Figure S2d,e), which was consistent with the MTT assay. Additionally, to validate the outcome of PAK2 depletion, we overexpressed PAK2 in BICR10 cells (Supplementary Figure S2f) and found significantly increased cell viability (Supplementary Figure S2g). Moreover, PAK2 depletion also led to the reduced expression of cell proliferation marker MKi67 at both transcript and protein levels (Fig. 2c, d, g, h). Collectively, these data indicate that PAK2 overexpression promotes the cellular proliferation and cell viability in HNC cells. Upon establishing the role of PAK2 in cellular proliferation, we investigated the role of PAK2 in cell-cycle progression using flow cytometry. Interestingly, PAK2 silencing significantly increased the cell population at G0/G1 phase in H157 (Fig. 2i) and H413 cells (Fig. 2k) and reduced the cell population in S phase. As the expression of Cyclin D1 (CCND1) has been shown to be critical for G1/S phase transition of cells^41^, we investigated the CCND1 expression upon PAK2 depletion. The PAK2-deficient H157 and H413 cells showed a remarkable decrease in CCND1 expression in comparison to control cells (Fig. 2j, l and Supplementary Figure S2h,i). Conclusively, these results demonstrate that PAK2 promotes cell-cycle progression by upregulating the CCND1 expression and promotes HNC cell proliferation.

### PAK2 depletion suppresses migration, invasion and colony formation of head and neck cancer cells and reduces chemotherapeutic resistance

**Fig. 3:**
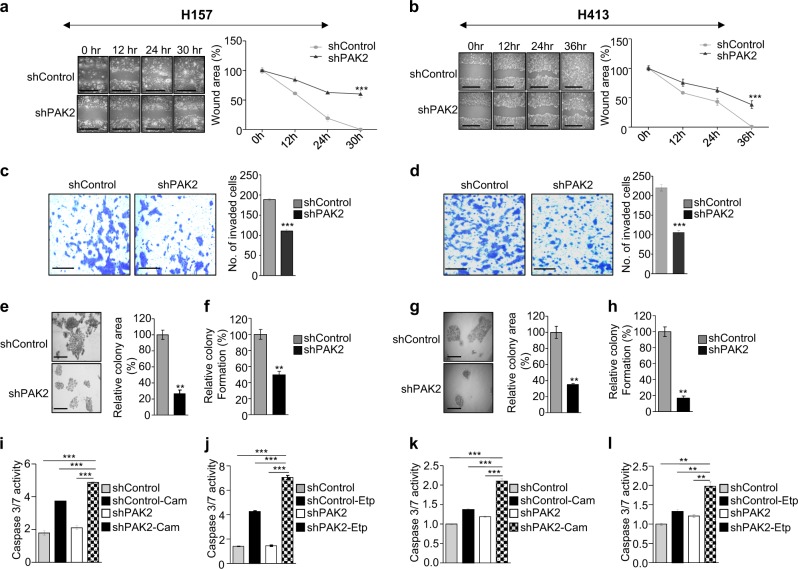
PAK2 depletion reduces in vitro tumorigenesis of head and neck cancer cell lines. PAK2 expression was depleted in two types of HNC cell lines (H157 and H413), and key cancer hallmarks were compared in PAK2-depleted and control cells. **a**, **b** Cell migration was analyzed through wound healing assay, (left) wound was observed under the microscope, scale bar 500 µm, and (right) quantification of wound width (*n* = 3). **c**, **d** The cell invasion was analyzed through Matrigel invasion assay, (left) single field of invaded cells was captured under the microscope, scale bar: 250 µm, and (right) invaded cells were counted at five different fields under the microscope (*n* = 3), **e**, **g** colony-forming tendency of cells was analyzed, (left) colony size was observed under the microscope, scale bar: 250 µm, and (right) quantification of colony size measured with ImageJ, **f**, **h** number of colonies were counted manually after crystal violet staining of the cells (*n* = 3). The caspase 3/7 activity of **i**, **j** H157 cells and **k**, **l** H413 cells was analyzed upon treatment with 5 µM camptothecin (Cam) and 50 µM etoposide (Etp) as indicated. Shown results are representative of three independent experiments and **f**, **h** are average of three independent experiments. Error bars shows mean values ± SD. Differences were considered statistically significant with **P < 0.01 and ***P < 0.001

To study the role of PAK2 in an in vitro tumorigenesis of HNC cells, the PAK2-depleted and control cells were subjected to wound healing, cell invasion and colony formation assay. Upon PAK2 depletion, we observed significantly reduced migration (Fig. 3a, b and Supplementary Figure S3a) and invasion (Fig. 3c, d and Supplementary Figure S3b). Similarly, the PAK2 overexpression in BICR10 cells significantly increased the migration and invasion of PAK2_OE cells as compared to the control cells (Supplementary Figure S3c,d). Sequentially, to assess the role of PAK2 in colony formation^42^ of HNC cells, we performed colony formation assay and observed reduced colony formation in PAK2-depleted cells as compared to control cells. The measurement of colony area, colony number (Fig. 3e–h and Supplementary Figure S3e,f) and colony density (Supplementary Figure S3g) of stained colonies showed a significant reduction in colony size as well as colony number upon PAK2 depletion. These results suggest that PAK2 affects migration, invasion and colony formation of HNC cells and thus helps in head and neck oncogenesis.

The chemotherapeutic resistance against the anticancer drug has been a challenge for cancer treatment. Interestingly, the Oncomine data analysis of HNC cells^43,44^ showed a negative correlation of PAK2 with anticancer drug treatment, wherein the expression of PAK2 was more in anticancer drug (paclitaxel and dimethyloxaloylglycine)-resistant cells in comparison to sensitive cells (Supplementary Figure S3j,k), which was consistent with the previous studies^45^. Sequentially, to investigate the role of PAK2 in chemotherapeutic drug-induced apoptosis in HNC cells, PAK2-depleted and control cells were treated with anticancer drugs, camptothecin and etoposide, which resulted in the increased apoptosis in PAK2-depleted cells (Fig. 3i–l and Supplementary Figure S3h-i). It was further validated by Annexin V–PI staining, wherein PAK2-depleted H157 and H413 cells showed higher staining of Annexin V and PI in comparison to control cells (Supplementary Figure S3l-o). Collectively, these data suggest that the higher expression of PAK2 provides resistance against chemotherapeutic drug-induced apoptosis and highlight the multidimensional role of PAK2 in head and neck tumorigenesis.

### PAK2 depletion inhibits activation of β-catenin and thereby reduces c-Myc expression and affects aerobic glycolysis via PKM2 downregulation

**Fig. 4:**
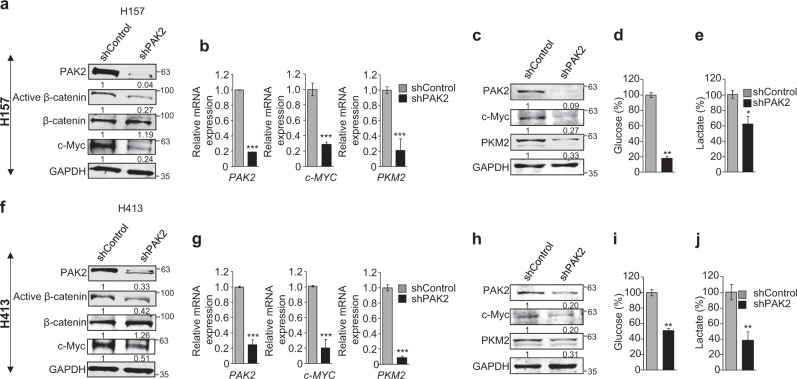
PAK2 depletion reduces active β-catenin (ABC) and downregulates c-Myc expression and inhibits Warburg effects. PAK2 expression was depleted in two types of HNC cells (H157 and H413) to understand its role in activation of β-catenin and c-Myc expression as well as in Warburg effect. **a**, **f** Protein expression of PAK2, active β-catenin, β-catenin and c-Myc upon PAK2 depletion (*n* = 3). **b**, **g** mRNA and **c**, **h** protein expression of PAK2, c-Myc and PKM2 were analyzed upon PAK2 depletion (*n* = 4). **d**, **i** The level of glucose uptake and **e**, **j** lactate production were analyzed upon PAK2 depletion (*n* = 3). Error bars shows mean values ± SD. Differences were considered statistically significant with *P < 0.05, **P < 0.01 and ***P < 0.001

The PAK2 has been proposed as an effector molecule of Rac1 signaling which is suggested to be necessary for the stabilization of activated β-catenin^46^. This indicates a possible and direct role of PAK2 in β-catenin-mediated signaling. Importantly, we observed a significant reduction of active β-catenin (ABC) upon PAK2 depletion; however, we did not observe any significant change in β-catenin expression (Fig. 4a, f). These findings were consistent with previous report^47^ which showed alteration of active β-catenin upon PAK2 depletion in schwannoma cells. This suggests that PAK2 affects the activation of β-catenin but not the expression of β-catenin. As the β-catenin activation is associated with regulation of downstream target genes, we hypothesized that the β-catenin signaling might be PAK2 dependent and PAK2 might also regulate the expression of Wnt/ABC target gene c-Myc^47,48^ in HNC cells. The c-Myc is overexpressed in various cancers including HNC^49^ and promotes cell proliferation, invasion and metastasis and is thereby considered as a molecular hallmark of cancer^50^. We investigated the dependency of PAK2-mediated head and neck tumorigenesis on c-Myc. Consequently, we validated the effects of ABC reduction on the c-Myc and found that the reduction of ABC upon PAK2 depletion correlates with significant downregulation of c-Myc (Fig. 4a, f), while, upon overexpressing PAK2 in BICR10, c-Myc expression was drastically increased (Supplementary Figure S2f). These data clearly establish the role of PAK2 in activation of β-catenin, which thereby upregulate c-Myc expression in HNC cells.

The c-Myc-mediated tumorigenesis is partly contributed by increased aerobic glycolysis or Warburg effect^51^, and as PKM2 plays an important role in enhancing the Warburg effect^52^, we investigated the effect of PAK2 depletion on the expression of PKM2. We observed significant downregulation of PKM2 (Fig. 4b, c, g, h) upon PAK2 depletion, while the overexpression of PAK2 showed elevated expression of PKM2 in comparison to control cells (Supplementary Figure S2f). The PKM2 expression is known to be associated with increased aerobic glycolysis, resulting in increased glucose uptake and lactate production in cancer cells^53^. Hence, we hypothesized that the PAK2 depletion might lead to a reversal of Warburg effect due to downregulation of PKM2. To test this hypothesis, we analyzed lactate production and glucose uptake in PAK2-depleted H157 and H413 cells. Interestingly, PAK2 depletion resulted in reduced lactate production and glucose uptake in HNC cells (Fig. 4d, e, i, j). Collectively, these results showed that PAK2 affects PKM2 expression and suggests an important role of PAK2 in cancer cell energy metabolisms.

### c-Myc complementation in PAK2-depleted cells restores PKM2 expression, Warburg effect and promotes head and neck carcinogenesis

**Fig. 5:**
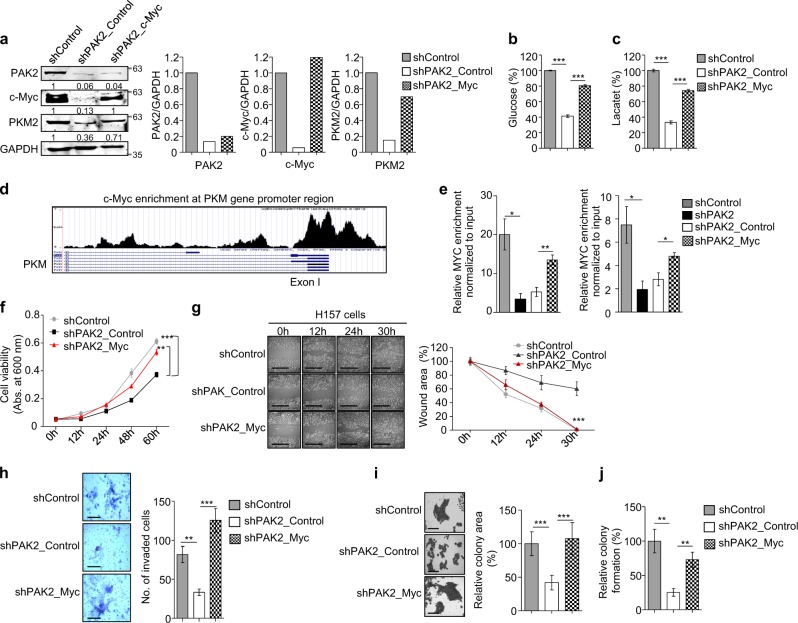
c-MYC complementation rescues the active proliferation. PAK2 expression was depleted in H157 cells, and c-Myc was ectopically overexpressed to understand the interdependency of PAK2 and c-Myc to induce oncogenesis. The key cancer hallmarks were analyzed upon c-Myc complementation in PAK2-deficient cells. **a** (Left) Protein expression of PAK2, c-Myc and PKM2 was analyzed upon c-Myc overexpression through immunoblotting (*n* = 3). (Right) Densitometric analysis of representative blot. **b** The level of glucose uptake and **c** lactate production was analyzed (*n* = 3). **d** Representative image shows the c-Myc enrichment at PKM gene promoter region, analyzed using UCSC genome browser. **e** Shown is the enrichment of c-Myc at PKM gene promoter region, analyzed with c-Myc ChIP (*n* = 3). **f** Relative cell proliferation was analyzed through MTT assay (*n* = 3). **g** Cell migration was analyzed through wound healing assay, (left) wound was observed under the microscope, scale bar: 500 µm, (right) quantification of wound width (*n* = 3), **h** cell invasion was analyzed upon crystal violet staining through Matrigel invasion assay, (left) single field of invaded cells was captured under the microscope, scale bar: 250 µm, (right) invaded cells were counted at five different fields under the microscope (*n* = 3), **i** colony-forming tendency of cells was analyzed, (left) colony sizes were observed under the microscope, scale bar: 250 µm, and (right) quantification of colony size measured with ImageJ. **j** Number of colonies were counted manually after crystal violet staining of the cells (*n* = 3). shPAK2_Control shPAK2+pAIP, shPAK2_Myc shPAK2+c-MYC. Error bars shows mean values ± SD. Differences were considered statistically significant with *P < 0.05, **P < 0.01 and ***P < 0.001

In the previous sections, we have shown that the PAK2 depletion is associated with the significantly reduced c-Myc and PKM2 expression (Fig. 4b, c, g, h). In order to validate the dependency of PAK2 on c-Myc for regulation of PKM2, we transiently overexpressed c-Myc in PAK2-deficient H157 cells (Supplementary Figure S4a). The overexpression of c-Myc in PAK2-depleted cells restored the expression of PKM2 in shPAK2_Myc cells as compared to shPAK2_Control cells (Fig. 5a and Supplementary Figure 4b, c). Sequentially, we also investigated the effect of c-Myc complementation and thereby PKM2 restoration on glucose metabolism. We observed a significantly increased glucose uptake and lactate production in shPAK2_Myc cells as compared to shPAK2_Control cells (Fig. 5b, c).

In order to understand the molecular mechanism of c-Myc-dependent PKM2 expression, we analyzed putative c-Myc-binding at *PKM* promoter region using Genomatix software suite (http://shop.genomatix.de/). The Genomatix software suit highlighted probable c-Myc binding sites at *PKM* promoter region (Supplementary Figure S4d). Similarly, analysis of published ChIP-seq data (GSM935320) with the UCSC genome browser revealed c-Myc enrichment at the *PKM* gene promoter region (Fig. 5d). Importantly, we observed a significant enrichment of c-Myc at the *PKM* gene promotor region, which is markedly reduced in PAK2-depleted cells (Fig. 5e), which correlates with PKM2 downregulation (Fig. 5a and Supplementary Figure 4c). Sequentially, upon c-Myc complementation, the c-Myc enrichment was restored at the *PKM* gene promoter (Fig. 5e), which further resulted in increased PKM2 expression (Fig. 5a and Supplementary Figure S4b,c). Moreover, c-Myc complementation also restored the expression of other glycolysis-associated genes (Supplementary Figure S4e). Conclusively, these results suggest that PAK2 depletion affects c-Myc–PKM2 expression and compromises the HNC cell energy metabolism; however, the rescue of Warburg effect upon complementation of c-Myc in PAK2-depleted cells indicates that PAK2–c-Myc–PKM2 axis plays an important role in cancer metabolism and thereby HNC development.

Furthermore, we also investigated the restoration of tumorigenic properties upon c-Myc complementation in PAK2-depleted HNC cells. The proliferation, migration, invasion and colony formation assay were analyzed in shPAK2_Myc and shPAK2_Control cells. Interestingly, we observed rescue of proliferation in shPAK2_Myc cells as compared to shPAK2_Control cells by MTT assay (Figure 5f). Additionally, the rescue of wound healing property was observed in shPAK2_Myc cells in comparison to shPAK2_Control cells, which suggest that PAK2-mediated c-Myc expression is critical for cellular proliferation and wound healing (Fig. 5g). Subsequently, we also performed invasion assay and colony formation assay, wherein we found that shPAK2_Myc cells showed restored invasion (Fig. 5h) and colony formation (Fig. 5i, j and Supplementary Figure S4f). Conclusively, these data suggest that PAK2 promotes HNC progression via regulating c-Myc expression.

### Global effect of PAK2 depletion in head and neck cancer cells

**Fig. 6:**
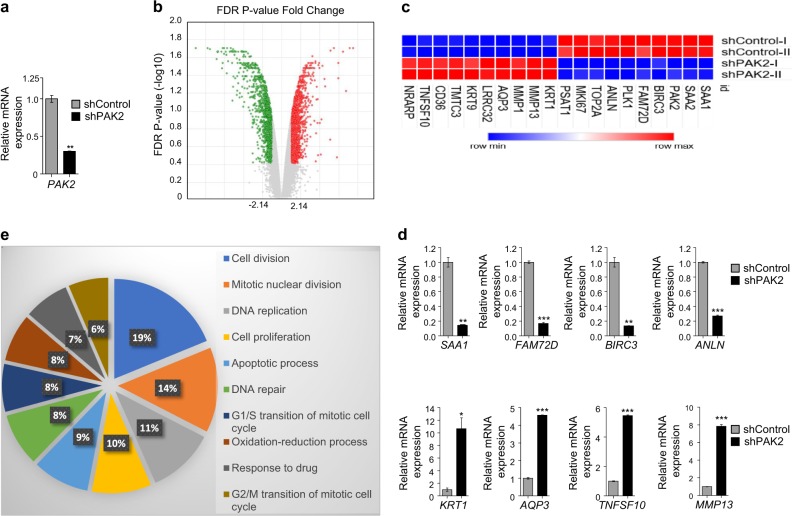
PAK2 depletion from head and neck cancer cells globally affects the expression of proliferation genes. The PAK2 was depleted in H157 cells and total mRNA was isolated for global analysis, human transcriptome array (HTA) 2.0. **a** The mRNA expression of *PAK2* was analyzed by qRT-PCR upon knockdown. **b** Globally differentially expressed genes were analyzed through volcano plot upon PAK2 depletion. **c** Heat map of top 10 up- and downregulated genes in head and neck cancer cells upon PAK2 knockdown. **d** The mRNA expression of differentially expressed genes, *serum amyloid A1* (*SAA1*), *family with sequence similarity 72 member D* (*FAM72D*), *baculoviral IAP repeat containing* 3 (*BIRC3*), *anillin actin binding protein* (*ANLN*), *keratin 1* (*KRT1*), *aquaporin 3* (*AQP3*), *TNF superfamily member 10* (*TNFSF10*) and *matrix metallopeptidase 13* (*MMP13*) were verified by quantitative real-time PCR and **e** Gene Ontology analysis of downregulated genes upon PAK2 depletion. Error bars shows mean values ± SD. Differences were considered statistically significant with *P < 0.05, **P < 0.01 and ***P < 0.001

Having shown that PAK2 promotes Warburg effect via expression of proto-oncogenes (c-Myc and PKM2) and helps in HNC progression, we investigated the PAK2-mediated global changes in PAK2-depleted H157 cells (Fig. 6a) by HTA 2.0. The PAK2 depletion in HNC cells resulted in differential expression of 831 genes (Fig. 6b). Furthermore, the top 20 differentially expressed genes (10 downregulated and 10 upregulated) were selected on the basis of significant false discovery rate *P* value criteria, and their expression was compared in both PAK2-depleted and control cells as shown in the heat map (Fig. 6c). The expression pattern of these genes was further cross-validated by analyzing their transcript level through qRT-PCR analysis (Fig. 6d and Supplementary Figure S5a). Additionally, gene ontology (GO) analysis of differentially expressed genes upon PAK2 depletion revealed an enrichment of genes involved in cell proliferation, DNA replication, cell cycle, apoptosis, nucleosome assembly, drug metabolism and other cellular processes (Fig. 6e). Moreover, the Kyoto Encyclopedia of Gene and Genomics (KEGG) analysis of proliferation pathway showed the downregulation of proliferation-associated genes upon PAK2 depletion (Supplementary Figure S5c). This implies the possible role of PAK2 in the expression of genes involved in the development of major hallmarks of cancer. Similarly, GO analysis of upregulated genes upon PAK2 depletion resulted in the enrichment of genes involved in cell adhesion, exosome processing, cell membrane integrity, calcium ion signaling and cellular filament organization (Supplementary Figure S5c), suggesting for a role of PAK2 in diverse cellular signaling. Collectively, these results suggest that PAK2 is a critical oncogene which brings about global changes in expression of cancer-associated genes.

### Data deposition

The HTA 2.0 array data reported in this report have been deposited in the GEO database, www.ncbi.nlm.nih.gov/geo (accession no. GSE113322).

## Discussion

Recent advancements in cancer epigenetics have shown the role of altered epigenetic events as key step in cancer initiation and progression^54^. The current understanding of global alterations in the epigenetic landscape during cancer initiation and progression warrant the identification of differentially expressed chromatin modifiers. Herein our study, we observed significant overexpression of a histone modifier PAK2 in HNC patients. The deregulated expression of PAK2 has been linked with different human malignancies^55^, and proposed to be positively associated with cellular transformation^56^ and proliferation^29^. However, the underlying molecular mechanism of PAK2-mediated oncogenesis remains poorly understood.

Our study demonstrated that the depletion of PAK2 compromised cellular proliferation. The molecular study highlighted that PAK2 depletion significantly reduced CCND1 expression (Fig. 2). Additionally, the expression of PAK2 in leukemic cells have been shown to affect invasion and angiogenesis^57,58^. Interestingly, the head and neck cancer showed a similar behavior of reduced migration and invasion upon PAK2 depletion, which established the fact that oncogenic activity of PAK2 is not cell type specific (Fig. 3).

Considering the fact that the PKM2 overexpression is associated with chemoresistance in various cancers^59,60^, the PAK2–c-Myc–PKM2 axis might be responsible for chemoresistance in HNC. Interestingly, we observed that PAK2 depletion reduced chemotherapeutic resistance in HNC cells, which further correlates with poor survival of HNC patients with higher expression of PAK2. This indicates that PAK2 might be used as a prognosis marker for HNC patients as proposed for gastric cancer^31^.

Furthermore, PAK2 is known to be an effector molecule of Rac1/Cdc42 signaling which is shown to be associated with activation of Wnt/ABC pathway^61^. We hypothesized that the PAK2 expression might be correlated with Wnt/ABC signaling and regulation of target gene expression such as c-Myc^62^. Notably, for the first time, we showed that the PAK2 depletion causes reduced ABC and thereby downregulation of c-Myc expression in HNC cells. The c-Myc, as an oncogene, is upregulated in various malignancies including HNC^63^ and c-Myc-deficient cells are unable to induce tumorigenesis^64^. Thus, it is likely that the regulation of c-Myc expression might have a possible role in PAK2-mediated head and neck oncogenesis.

Furthermore, the role of c-Myc in PAK2-mediated oncogenesis was validated by c-Myc complementation in PAK2-depleted HNC cells, which resulted in complete restoration of oncogenic potential as reflected by an increase in cell proliferation, invasion, and migration (Fig. 5). These results strengthen our hypothesis that the PAK2-mediated head and neck oncogenesis is dependent on the oncogenic role of c-Myc.

Additionally, recent reports have shown that c-Myc regulates the expression of genes involved in glycolysis^65–67^. Interestingly, for the first time, we showed that the PAK2 also regulates the expression of aerobic glycolysis-associated genes via regulation of c-Myc, wherein PAK2 depletion resulted in significantly reduced expression of glucose transporter 1 (GLUT1), lactate dehydrogenase A and B (LDHA and LDHB) and enolase 1 (ENO1). The enhanced expression of these genes is known to be positively associated with cancer progression^66^.

**Fig. 7:**
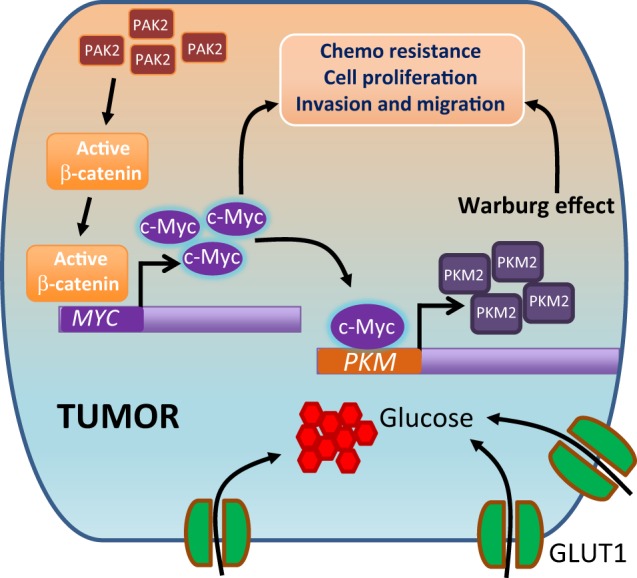
Schematic model. Elevated expression of PAK2 leads to increased activation of β-catenin and promotes the expression of Wnt/ABC target gene, c-Myc. The upregulated expression of c-Myc promotes Warburg effect via promoting PKM2 expression as well as other glycolytic genes and promotes tumorigenesis

Moreover, the correlation of c-Myc expression with upregulated PKM2 in various cancers^67,68^ highlights a possible role of c-Myc in PKM2 expression. However, the molecular mechanism of PKM2 upregulation by c-Myc remains to be elucidated. The expression of PKM2 has been shown to promote the Warburg effect, proliferation and tumor growth^69,70^. Interestingly, we observed that the depletion of PAK2 showed a remarkable reduction in PKM2 expression and thereby reduced Warburg effect, which was rescued by c-Myc complementation in PAK2-deficient HNC cells. Importantly our result shows the molecular regulation of PAK2–c-Myc–PKM2 axis and upregulation of this axis enhanced the glucose uptake and cell proliferation (Fig. 7). This finding was in coherence with the earlier studies that established a positive correlation of PKM2 with cyclin D1^71^ and Ki67^72^ expression. Moreover, we unravel the c-Myc-dependent regulation of PKM2 expression, wherein we identified c-Myc binding site at *PKM* gene promoter. The reduced enrichment of c-Myc on *PKM* gene promoter region in PAK2-depleted cells highlighted the role of c-Myc in PKM2 expression which was validated by c-Myc complementation, which led to increased enrichment of c-Myc on *PKM* promoter region and thereby increased PKM2 expression. This suggests that c-Myc-dependent PKM2 expression plays an important role in PAK2-mediated oncogenesis.

Finally, our global transcriptome analysis showed that in addition to c-Myc and PKM2, PAK2 also regulates the expression of several genes, having an important role in cell proliferation, DNA repair, apoptosis and cellular transformation. The positive correlation of proliferation-associated genes with PAK2 emphasizes the importance of PAK2 in HNC growth and progression.

The present study highlights the undiscovered role of PAK2 in head and neck oncogenesis, wherein we showed that the elevated expression of PAK2 promotes head and neck cancer growth and provides chemotherapeutic resistance. Further molecular analysis identified that PAK2 upregulates c-Myc and promotes c-Myc-dependent PKM2 expression. Conclusively, we showed that the PAK2–c-Myc–PKM2 axis is critical for HNC progression and may provide an alternative strategy for multiple drug targets.

## Electronic supplementary material

Supplementary Figure S1

Supplementary Figure S2

Supplementary Figure S3

Supplementary Figure S4

Supplementary Figure S5

Supplementary Figure legends

Supplementary Table S1

Supplementary Table S2
