# Supplementary material for: PAK2–c-Myc–PKM2 axis plays an essential role in head and neck oncogenesis via regulating Warburg effect
Source: Cell Death Dis. 2018 Aug 1;9(8):825. doi: 10.1038/s41419-018-0887-0 (PMC6070504; doi:10.1038/s41419-018-0887-0)
Supplement: Supplementary file 1 — Supplementary Figure S1 [file 41419_2018_887_MOESM1_ESM.pptx]

## Slide 1
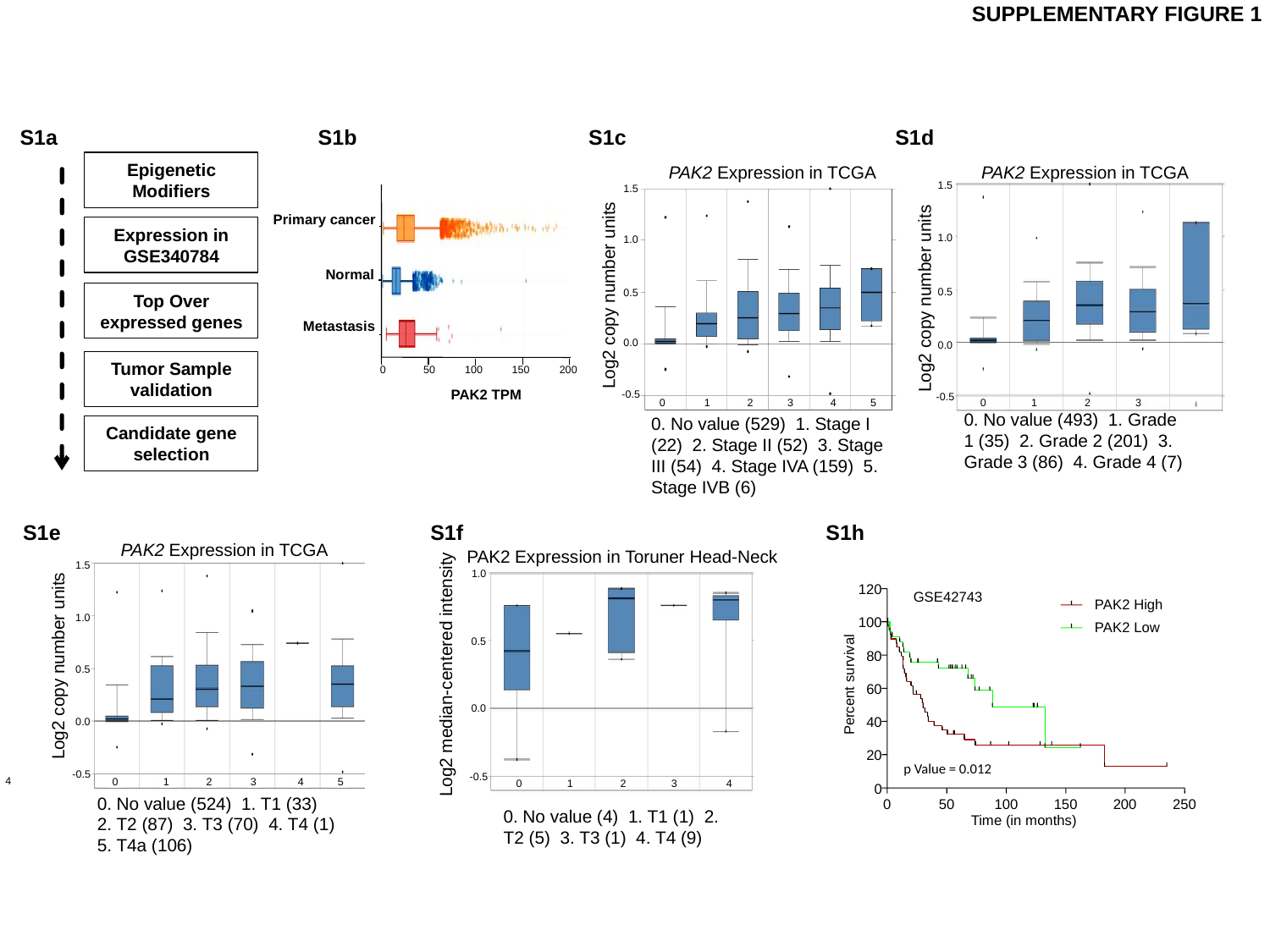

SUPPLEMENTARY FIGURE 1
S1a
Epigenetic Modifiers
Expression in GSE340784
Top Over expressed genes
Tumor Sample validation
Candidate gene selection
S1b
0
50
100
150
200
Primary cancer
Normal
Metastasis
PAK2 TPM
S1c
PAK2 Expression in TCGA
0. No value (529) 1. Stage I (22) 2. Stage II (52) 3. Stage III (54) 4. Stage IVA (159) 5. Stage IVB (6)
Log2 copy number units
1.5
1.0
0.5
0.0
-0.5
0
1
2
3
4
S1d
PAK2 Expression in TCGA
0. No value (493) 1. Grade 1 (35) 2. Grade 2 (201) 3. Grade 3 (86) 4. Grade 4 (7)
Log2 copy number units
1.5
1.0
0.5
0.0
-0.5
5
0
1
2
3
S1e
PAK2 Expression in TCGA
0. No value (524) 1. T1 (33) 2. T2 (87) 3. T3 (70) 4. T4 (1) 5. T4a (106)
Log2 copy number units
1.5
1.0
0.5
0.0
-0.5
4
0
1
2
3
4
5
S1f
PAK2 Expression in Toruner Head-Neck
0. No value (4) 1. T1 (1) 2. T2 (5) 3. T3 (1) 4. T4 (9)
Log2 median-centered intensity
1.0
0.5
0.0
-0.5
0
1
2
3
4
S1h
120
GSE42743
100
80
Percent survival
60
40
20
0
0
50
100
150
200
250
Time (in months)
PAK2 High
PAK2 Low
p Value = 0.012

## Slide 2
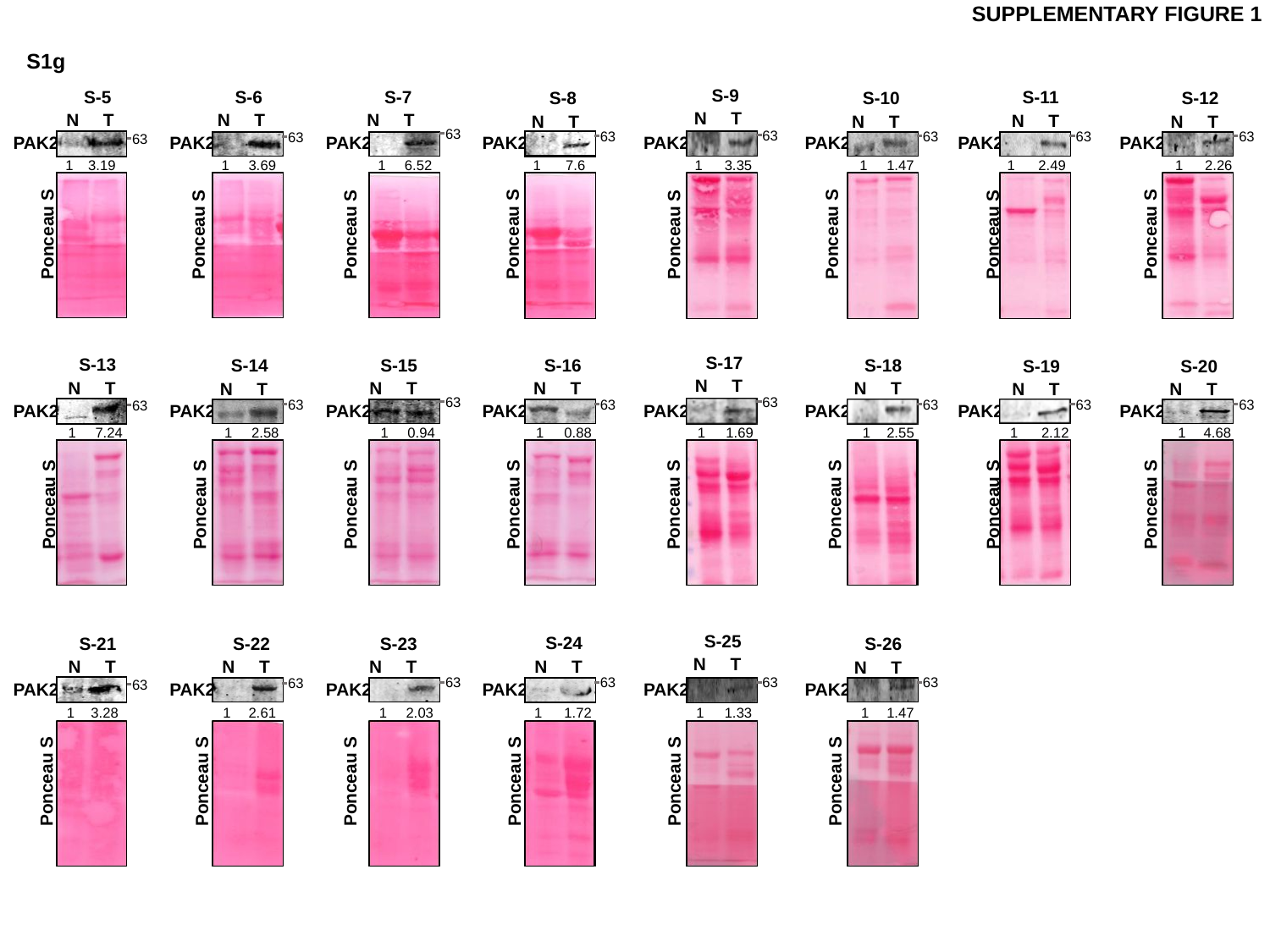

SUPPLEMENTARY FIGURE 1
S1g
S-9
N
T
63
PAK2
Ponceau S
S-7
N
T
63
PAK2
Ponceau S
S-5
N
T
63
PAK2
Ponceau S
S-6
N
T
63
PAK2
Ponceau S
S-11
N
T
63
PAK2
Ponceau S
S-10
N
T
63
PAK2
Ponceau S
S-12
N
T
63
PAK2
Ponceau S
S-8
N
T
63
PAK2
Ponceau S
S-17
N
T
63
PAK2
Ponceau S
S-13
N
T
63
PAK2
Ponceau S
S-16
N
T
63
PAK2
Ponceau S
S-18
N
T
63
PAK2
Ponceau S
S-15
N
T
63
PAK2
Ponceau S
S-14
N
T
63
PAK2
Ponceau S
S-19
N
T
63
PAK2
Ponceau S
S-20
N
T
63
PAK2
Ponceau S
S-25
N
T
63
PAK2
Ponceau S
S-24
N
T
63
PAK2
Ponceau S
S-22
N
T
63
PAK2
Ponceau S
S-23
N
T
63
PAK2
Ponceau S
S-21
N
T
63
PAK2
Ponceau S
S-26
N
T
63
PAK2
Ponceau S
1
1
1
1
1
1
1
1
3.19
3.69
6.52
7.6
3.35
1.47
2.49
2.26
1
1
1
1
1
1
1
1
7.24
2.58
0.94
0.88
1.69
2.55
2.12
4.68
1
1
1
1
1
1
3.28
2.61
2.03
1.72
1.33
1.47
