# Supplementary material for: PAK2–c-Myc–PKM2 axis plays an essential role in head and neck oncogenesis via regulating Warburg effect
Source: Cell Death Dis. 2018 Aug 1;9(8):825. doi: 10.1038/s41419-018-0887-0 (PMC6070504; doi:10.1038/s41419-018-0887-0)
Supplement: Supplementary file 2 — Supplementary Figure S2 [file 41419_2018_887_MOESM2_ESM.pptx]

## Slide 1
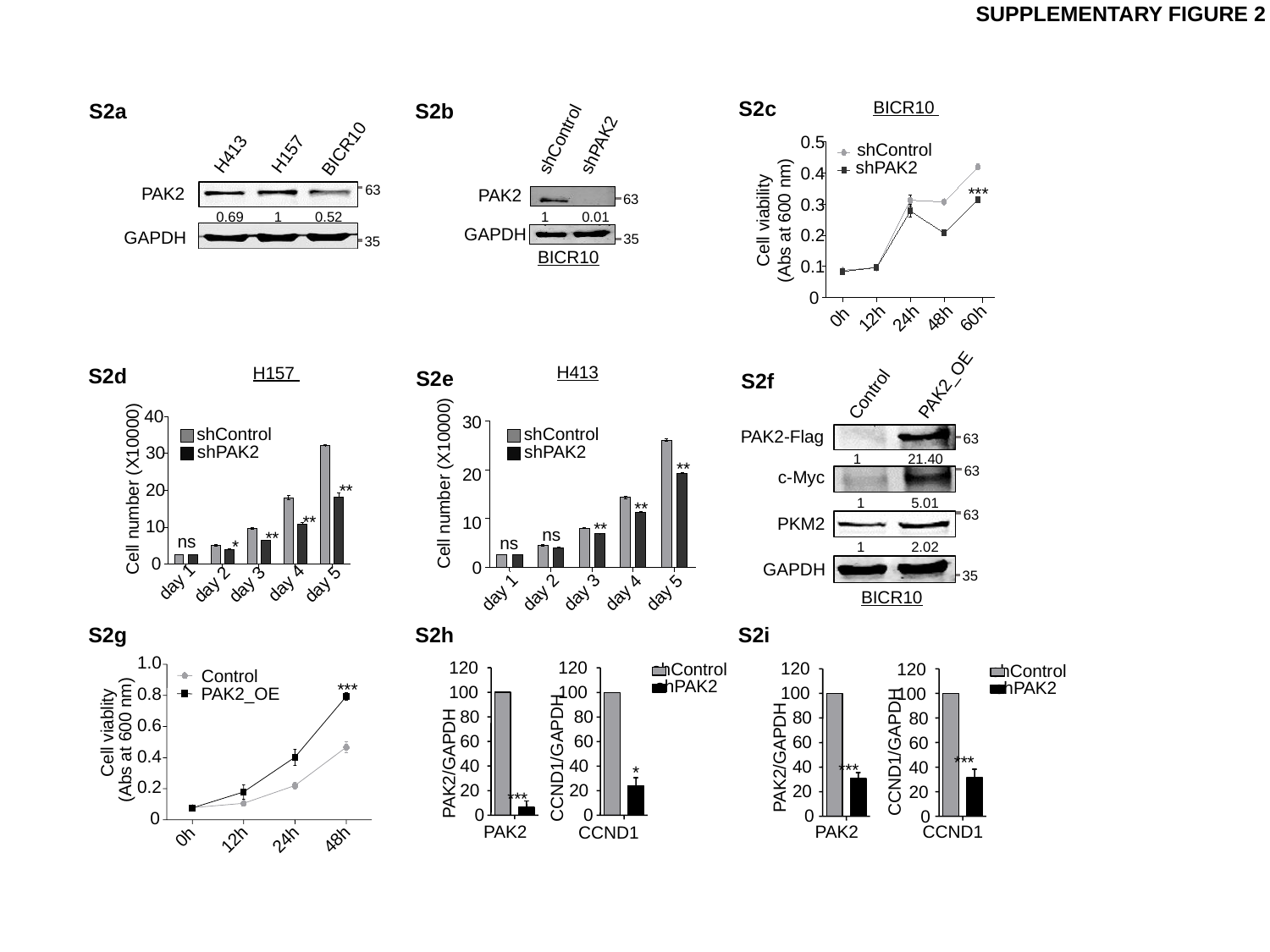

SUPPLEMENTARY FIGURE 2
shControl
shPAK2
BICR10
PAK2
GAPDH
S2b
63
35
1
0.01
S2c
BICR10
0.5
shControl
shPAK2
0.4
0.3
Cell viability
(Abs at 600 nm)
0.2
0.1
0
0h
60h
12h
24h
48h
***
S2a
H413
H157
63
PAK2
GAPDH
35
0.69
1
0.52
PAK2_OE
Control
PAK2-Flag
c-Myc
GAPDH
PKM2
1
21.40
1
5.01
1
2.02
BICR10
63
63
63
35
H413
30
shControl
shPAK2
**
20
Cell number (X10000)
**
**
10
0
day 2
day 3
day 1
day 5
day 4
ns
ns
H157
S2d
40
shControl
shPAK2
30
**
Cell number (X10000)
20
**
10
**
*
0
day 1
day 4
day 2
day 3
day 5
ns
S2e
S2f
S2g
S2h
S2i
1.0
Control
PAK2_OE
0.8
0.6
Cell viablity
(Abs at 600 nm)
0.4
0.2
0
0h
12h
24h
48h
***
120
100
80
60
40
PAK2/GAPDH
20
0
PAK2
120
shControl
shPAK2
100
80
60
CCND1/GAPDH
40
**
20
0
CCND1
120
100
80
60
PAK2/GAPDH
40
20
0
PAK2
***
120
shControl
shPAK2
100
80
60
***
CCND1/GAPDH
40
20
0
CCND1
***
BICR10
