# Supplementary material for: PAK2–c-Myc–PKM2 axis plays an essential role in head and neck oncogenesis via regulating Warburg effect
Source: Cell Death Dis. 2018 Aug 1;9(8):825. doi: 10.1038/s41419-018-0887-0 (PMC6070504; doi:10.1038/s41419-018-0887-0)
Supplement: Supplementary file 3 — Supplementary Figure S3 [file 41419_2018_887_MOESM3_ESM.pptx]

## Slide 1
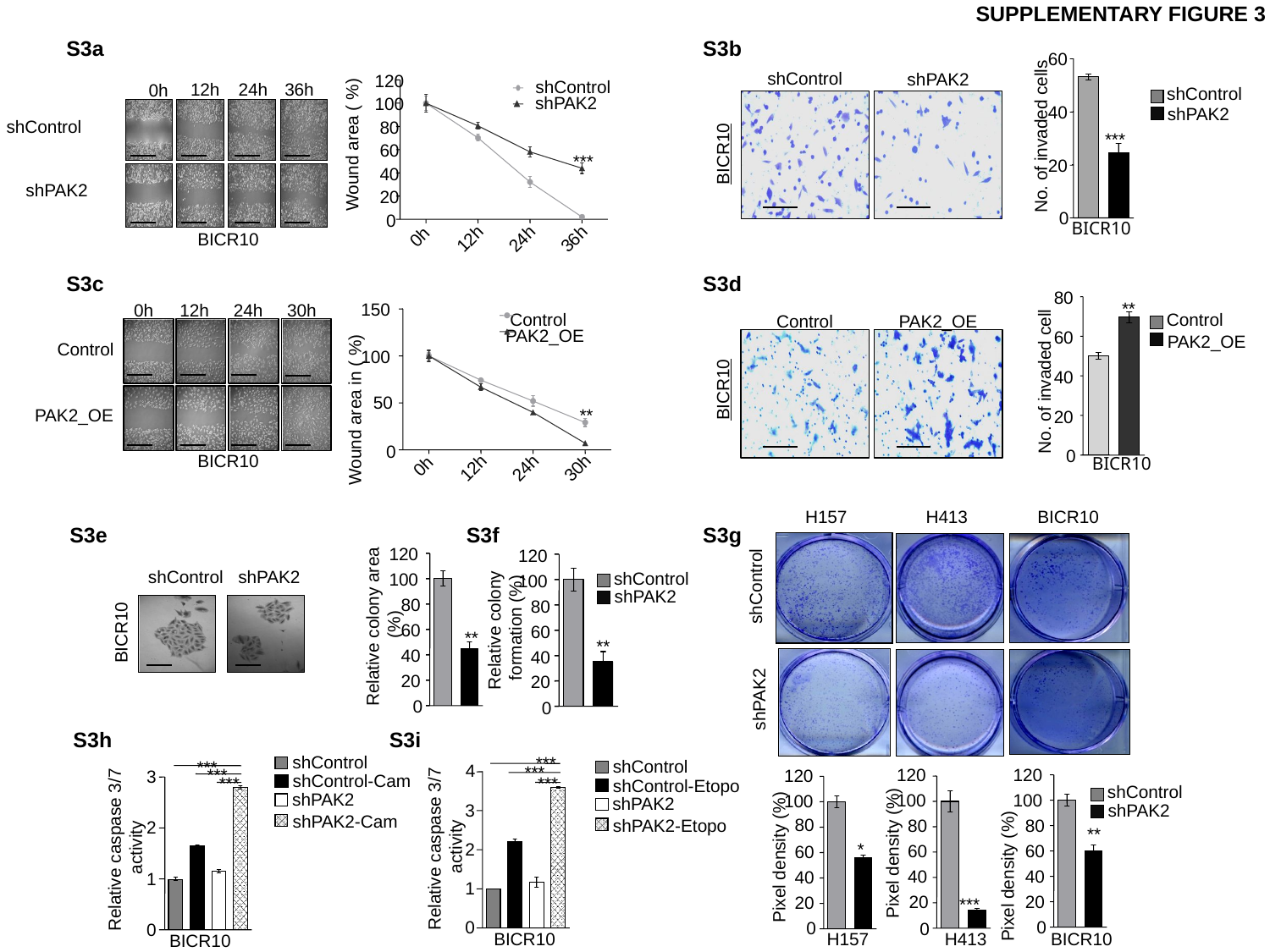

SUPPLEMENTARY FIGURE 3
S3a
S3b
60
40
No. of invaded cells
20
0
***
shControl
shPAK2
shControl
shPAK2
BICR10
120
shControl
shPAK2
100
80
Wound area ( %)
60
***
40
20
0
0h
12h
24h
36h
12h
24h
36h
shControl
0h
shPAK2
BICR10
BICR10
S3c
S3d
80
60
No. of invaded cell
40
20
0
**
Control
PAK2_OE
Control
PAK2_OE
BICR10
BICR10
0h
12h
24h
30h
Control
PAK2_OE
150
Control
PAK2_OE
100
50
Wound area in ( %)
0
0h
12h
24h
30h
**
H157
H413
BICR10
shControl
shPAK2
120
100
80
60
40
Pixel density (%)
20
0
120
100
80
60
Pixel density (%)
40
20
0
***
120
100
80
60
Pixel density (%)
40
20
0
*
H157
H413
BICR10
shControl
shPAK2
**
S3e
S3f
S3g
120
100
80
Relative colony area
(%)
60
40
20
0
120
100
80
Relative colony
formation (%)
60
40
20
0
**
shControl
shPAK2
BICR10
shControl
shPAK2
**
S3h
S3i
***
***
shControl
shControl-Etopo
shPAK2
shPAK2-Etopo
4
***
3
Relative caspase 3/7
activity
2
1
0
***
shControl
shControl-Cam
shPAK2
shPAK2-Cam
***
3
2
Relative caspase 3/7
activity
1
0
***
BICR10
BICR10
BICR10

## Slide 2
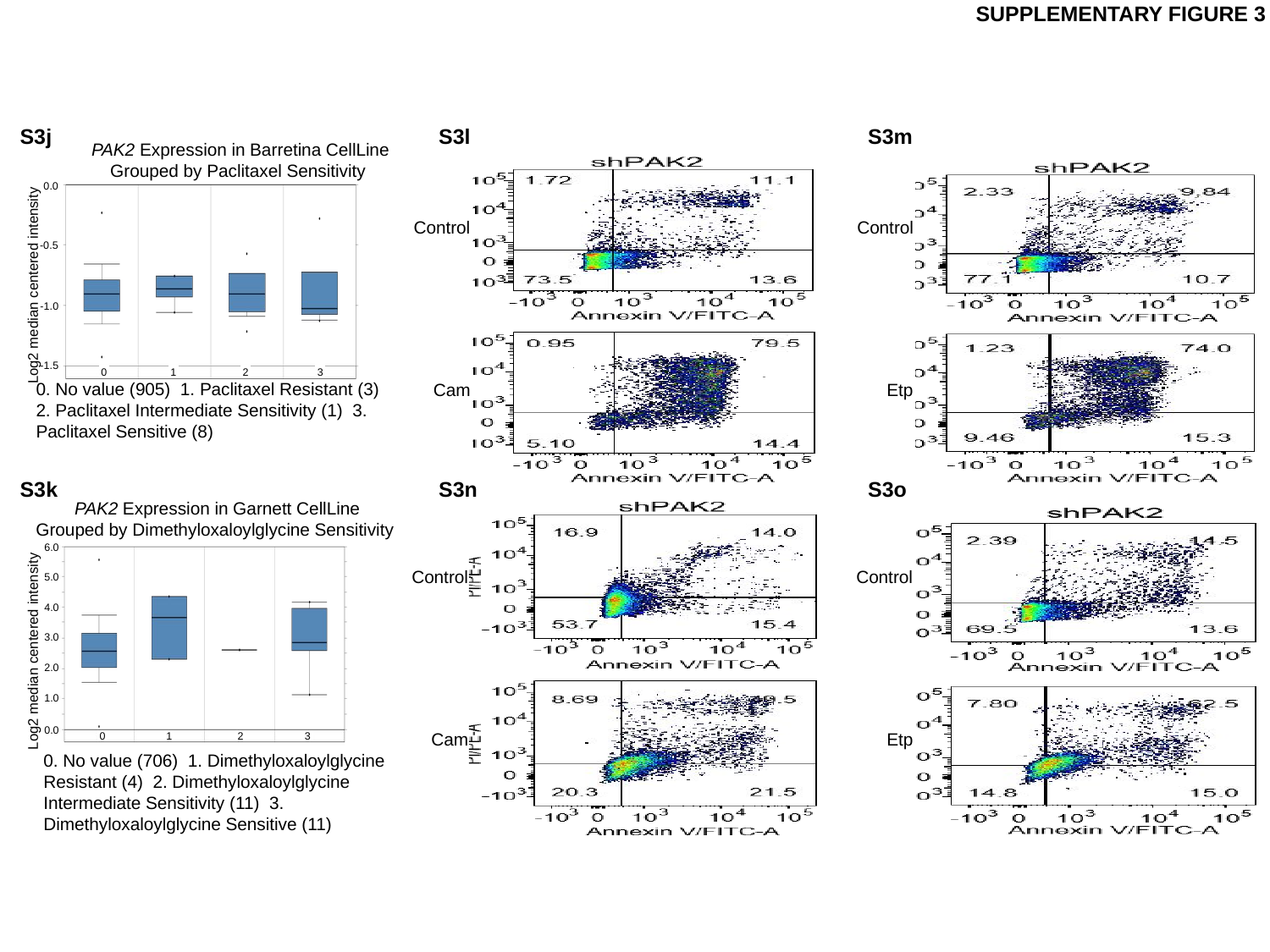

SUPPLEMENTARY FIGURE 3
S3j
PAK2 Expression in Barretina CellLine Grouped by Paclitaxel Sensitivity
0. No value (905) 1. Paclitaxel Resistant (3) 2. Paclitaxel Intermediate Sensitivity (1) 3. Paclitaxel Sensitive (8)
0.0
-0.5
-1.0
-1.5
Log2 median centered intensity
0
1
2
3
S3k
PAK2 Expression in Garnett CellLine
Grouped by Dimethyloxaloylglycine Sensitivity
0. No value (706) 1. Dimethyloxaloylglycine Resistant (4) 2. Dimethyloxaloylglycine Intermediate Sensitivity (11) 3. Dimethyloxaloylglycine Sensitive (11)
6.0
2.0
0.0
5.0
4.0
3.0
Log2 median centered intensity
1.0
0
1
2
3
S3l
S3m
Control
Control
Cam
Etp
S3n
S3o
Control
Control
Cam
Etp
