# Supplementary material for: PAK2–c-Myc–PKM2 axis plays an essential role in head and neck oncogenesis via regulating Warburg effect
Source: Cell Death Dis. 2018 Aug 1;9(8):825. doi: 10.1038/s41419-018-0887-0 (PMC6070504; doi:10.1038/s41419-018-0887-0)
Supplement: Supplementary file 4 — Supplementary Figure S4 [file 41419_2018_887_MOESM4_ESM.pptx]

## Slide 1
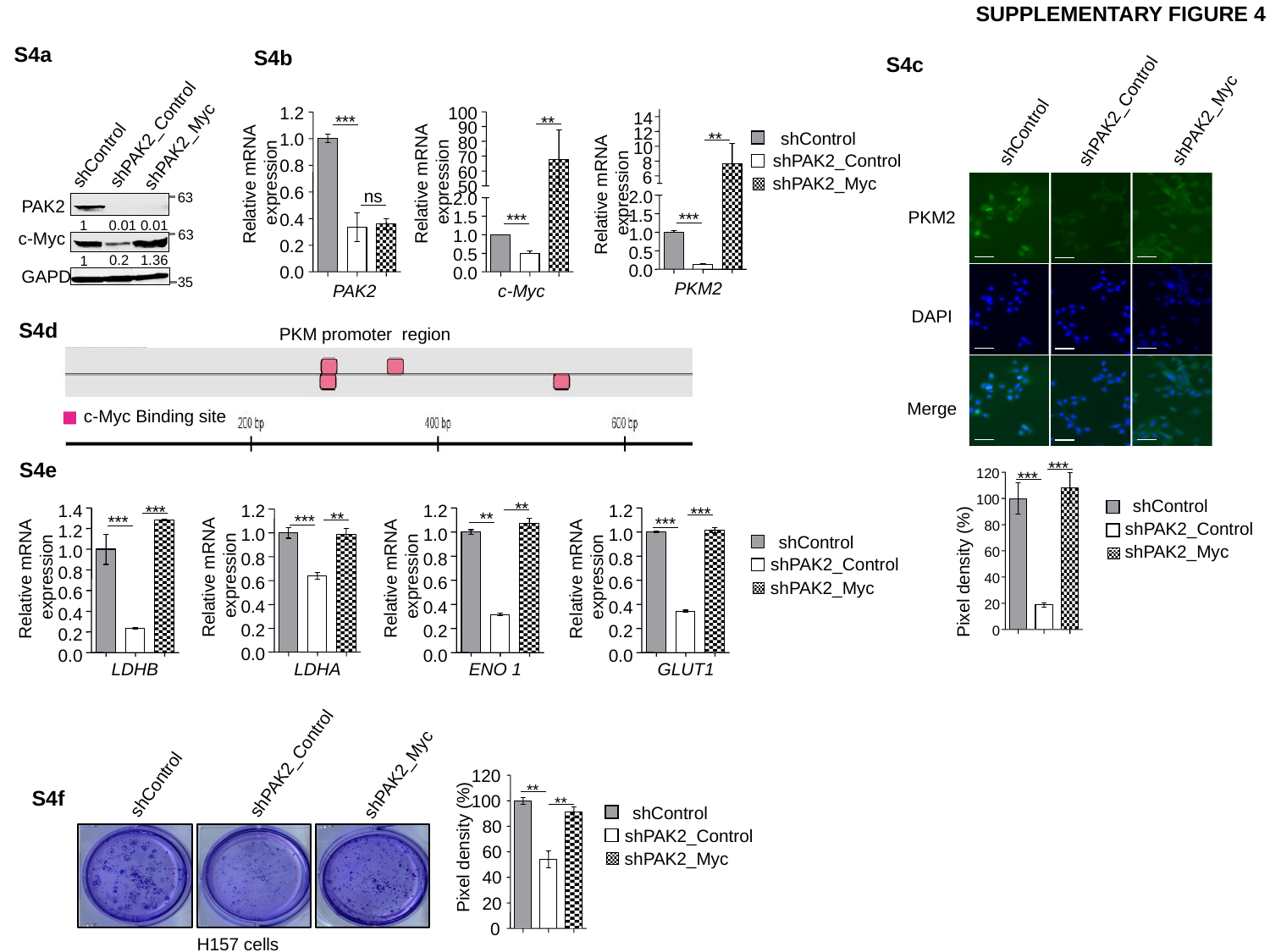

SUPPLEMENTARY FIGURE 4
PKM2
Merge
DAPI
shControl
shPAK2_Control
shPAK2_Myc
***
***
120
100
80
60
Pixel density (%)
40
20
0
shControl
shPAK2_Control
shPAK2_Myc
S4a
S4b
14
12
10
8
6
Relative mRNA
expression
2.0
1.5
1.0
0.5
0.0
PKM2
1.2
1.0
0.8
Relative mRNA
expression
0.6
0.4
0.2
0.0
PAK2
100
90
80
70
Relative mRNA
expression
60
50
2.0
1.5
1.0
0.5
0.0
c-Myc
***
ns
**
***
**
***
shControl
shPAK2_Control
shPAK2_Myc
S4c
shPAK2_Control
shPAK2_Myc
shControl
63
PAK2
c-Myc
GAPDH
S4d
PKM promoter region
c-Myc Binding site
S4e
1.4
1.2
1.0
Relative mRNA
expression
0.8
0.6
0.4
0.2
0.0
LDHB
***
***
1.2
1.0
0.8
Relative mRNA
expression
0.6
0.4
0.2
0.0
LDHA
**
***
1.2
1.0
0.8
Relative mRNA
expression
0.6
0.4
0.2
0.0
ENO 1
**
**
***
***
1.2
1.0
0.8
Relative mRNA
expression
0.6
0.4
0.2
0.0
GLUT1
shControl
shPAK2_Control
shPAK2_Myc
shPAK2_Control
shPAK2_Myc
shControl
H157 cells
S4f
120
**
**
100
80
Pixel density (%)
60
40
20
0
shControl
shPAK2_Control
shPAK2_Myc
0.01
0.2
1
1
1.36
63
35
0.01
