# Supplementary material for: PAK2–c-Myc–PKM2 axis plays an essential role in head and neck oncogenesis via regulating Warburg effect
Source: Cell Death Dis. 2018 Aug 1;9(8):825. doi: 10.1038/s41419-018-0887-0 (PMC6070504; doi:10.1038/s41419-018-0887-0)
Supplement: Supplementary file 5 — Supplementary Figure S5 [file 41419_2018_887_MOESM5_ESM.pptx]

## Slide 1
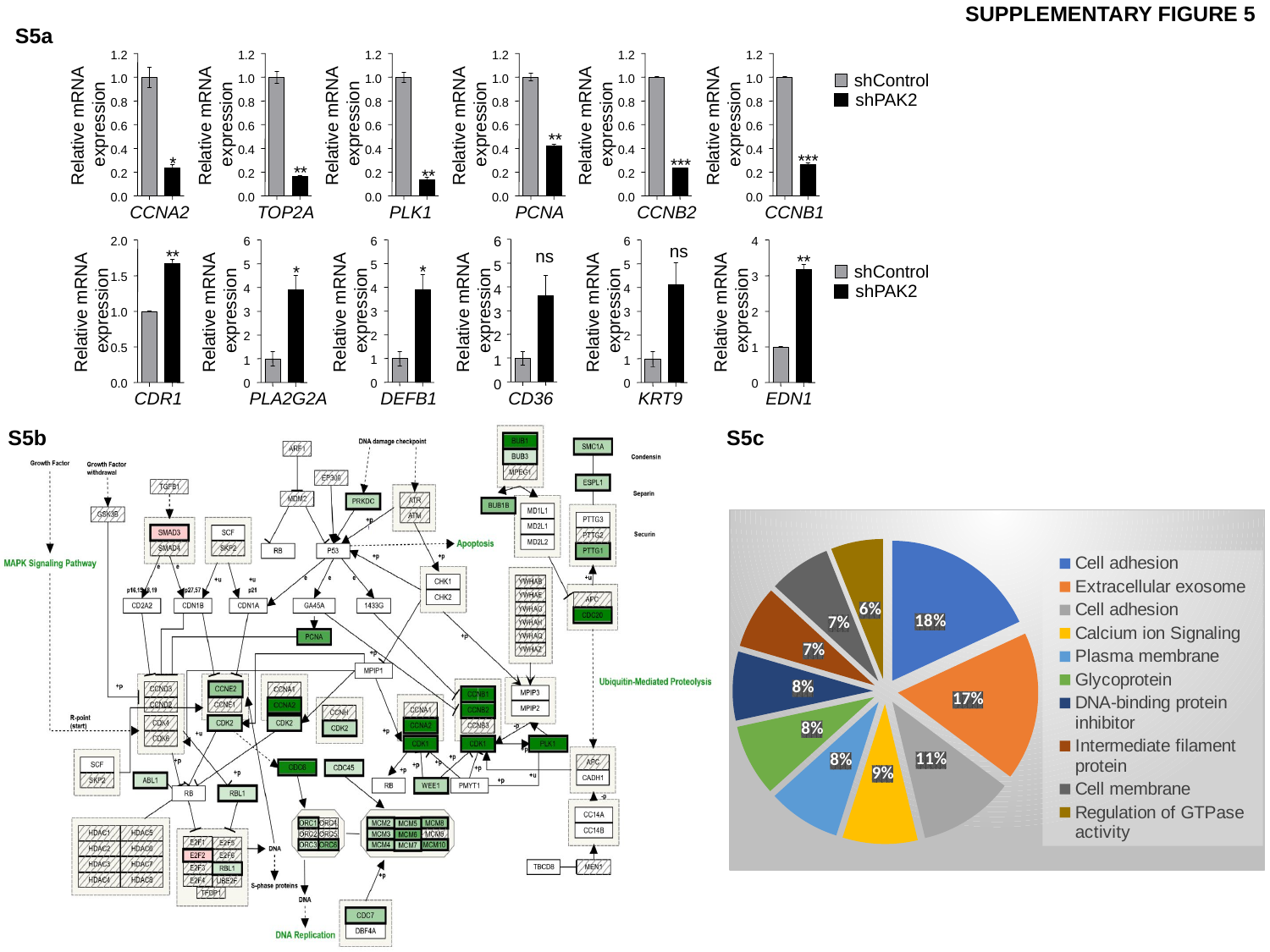

SUPPLEMENTARY FIGURE 5
1.2
1.0
0.8
Relative mRNA
expression
0.6
0.4
0.2
0.0
CCNA2
1.2
1.0
0.8
Relative mRNA
expression
0.6
0.4
0.2
0.0
TOP2A
1.2
1.0
0.8
Relative mRNA
expression
0.6
0.4
0.2
0.0
PLK1
1.2
1.0
0.8
Relative mRNA
expression
0.6
0.4
0.2
0.0
PCNA
1.2
1.0
0.8
Relative mRNA
expression
0.6
0.4
0.2
0.0
CCNB2
1.2
1.0
0.8
Relative mRNA
expression
0.6
0.4
0.2
0.0
CCNB1
**
***
*
***
**
**
shControl
shPAK2
6
5
4
Relative mRNA
expression
3
2
1
0
CD36
6
5
4
Relative mRNA
expression
3
2
1
0
DEFB1
2.0
1.5
Relative mRNA
expression
1.0
0.5
0.0
CDR1
6
5
4
Relative mRNA
expression
3
2
1
0
PLA2G2A
6
5
4
Relative mRNA
expression
3
2
1
0
KRT9
4
3
Relative mRNA
expression
2
1
0
EDN1
ns
ns
**
**
*
*
shControl
shPAK2
S5a
S5b
S5c
### Chart
| Category | |
|---|---|
| Cell adhesion | 18.06818181818182 |
| Extracellular exosome | 17.15909090909091 |
| Cell adhesion | 11.022727272727273 |
| Calcium ion Signaling | 8.636363636363637 |
| Plasma membrane | 8.409090909090908 |
| Glycoprotein | 8.295454545454547 |
| DNA-binding protein inhibitor | 7.954545454545454 |
| Intermediate filament protein | 7.2727272727272725 |
| Cell membrane | 7.159090909090909 |
| Regulation of GTPase activity | 6.0227272727272725 |
