# Supplementary material for: PAK2–c-Myc–PKM2 axis plays an essential role in head and neck oncogenesis via regulating Warburg effect
Source: Cell Death Dis. 2018 Aug 1;9(8):825. doi: 10.1038/s41419-018-0887-0 (PMC6070504; doi:10.1038/s41419-018-0887-0)
Supplement: Supplementary file 6 — Supplementary Figure legends [file 41419_2018_887_MOESM6_ESM.docx]

**Supplementary Figure Legends**

**Supplementary Figure S1: (S1a)** Graphical method of selecting the candidate HMs. **(S1b)** The RNAseq data of PAK2 expression in primary cancer, normal tissue and metastatic tissues of HNC, analyzed using MiPanda database (<http://www.mipanda.org>). **(S1c-f)** Expression analysis of PAK2 in different grade and stage of HNC tumor samples of TCGA analysis, analyzed by using Oncomine database **(S1c)** Shows the stage wise expression of PAK2 in HNC tumor samples. **(S1d)** The tumor grade specific expression of PAK2 in HNC tumor samples. **(S1e)** The expression of PAK2 with in different T-stage of HNC tumor samples. **(S1f)** PAK2 expression in HNC tumor samples with different T-staging extracted from Toruner Head-Neck database, analyzed using Oncomine database. **(S1g)** PAK2 protein expression analysis in HNC tumor and paired normal samples. **(S1h)** The survival data of HNC patient obtained from Gene Expression Omnibus (GSE42743) and Kaplan-Meier survival curve analysis between group of low PAK2 and high PAK2 expressing patients. TPM: Transcript per million. Error bars shows mean values ± SD. *P*-Value <0.05 considered as significant.

**Supplementary Figure S2:** The PAK2 protein expression was analyzed in HNC cells **(S2a)** H413, H157, BICR10. The PAK2 was depleted in BICR10 cells (n=3). **(S2b)** PAK2 immunoblotting in PAK2 depleted BICR10 cells (n=3). **(S2c)** Relative cell proliferation of BICR10 cells was analyzed through MTT assay (n=3). Cell-proliferation of **(S2d)** H157 and **(e)** H413 cells was analyzed through counting the number of cells for five days as indicated (n=3). **(S2f)** Immunoblotting of flag-PAK2, c-Myc, PKM2, GAPDH (n=3) and **(S2g)** relative cell proliferation of PAK2 overexpressing BICR10 cell was analyzed through MTT (n=3). Average of densitometric analysis of CCND1 of three independent experiments upon PAK2 depletion in **(S2h)** H157 and **(S2i)** H413. Error bars shows mean values ± SD. Differences were considered statistically significant with a *p-value<0.05, **p-value<0.01 and ***p-value<0.001.

**Supplementary Figure S3:** The PAK2 was depleted in BICR10, H157 and H413 HNC cells. **(S3a)** BICR10 cell-migration was analyzed through wound healing assay, **(left)** wound was observed under the microscope, scale bar: 500 µm, **(right)** quantification of wound width (n=3). **(S3b)** BICR10 cell-invasion was analyzed through Matrigel-invasion assay **(left)** single field of invaded cells was captured under the microscope, scale bar: 250 µm, **(right)** invaded cells were counted at five different field under the microscope (n=3). **(S3c)** PAK2 overexpressing BICR10 cells was analyzed through wound healing assay, **(left)** wound was observed under microscope, scale bar: 500 µm, **(right)** quantification of wound width (n=2)**. (S3d)** PAK2 overexpressing BICR10 cell-invasion was analyzed through Matrigel-invasion assay **(left)** single field of invaded cells was captured under the microscope, scale bar: 250 µm, **(right)** invaded cells were counted at five different field under the microscope (n=2). **(S3e)** Colony-forming tendency of cells was analyzed, **(left)** colony size was observed under the microscope, scale bar: 200 µm, and **(right)** quantification of colony size measured with ImageJ, **(S3f)** number of colonies were counted manually after crystal violet staining of the cells (n=3), **(S3g)** pixel density of colonies was measured after staining the cells with crystal violet stain by using ImageJ software **(top)** Scanned image H157, H413, BICR10 after crystal violet staining **(bottom)** bar graph shows relative pixel density quantification performed using ImageJ software. Caspase 3/7 activity of PAK2-depleted and control BICR10 HNC cells was observed upon treatment with **(S3h-i)** 5µM camptothecin (Cam) and 50µM etoposide (Etp) as indicated (n=3). **(S3j-k)** The differential expression of PAK2 in anticancer drug treated HNC cells analyzed using Oncomine database. Annexin V-PI staining of PAK2 depleted and control HNC cells upon anticancer drug treatment, **(S3l-m)** 5µM camptothecin (Cam) and 50 µM etoposide (Etp) in H157, **(S3n-o)** 5µM camptothecin (Cam) and 50µM etoposide (Etp) in H413 (n=3). Error bars shows mean values ± SD. Differences were considered statistically significant with a *p-value<0.05, **p-value<0.01 and ***p-value<0.001.

**Supplementary Figure S4:** Effect of c-Myc complementation in PAK2 depleted HNC cells, H157 **(S4a)** Protein expression of PAK2 and c-Myc analyzed by immunoblotting of respective protein (n=3). **(S4b)** The qRT-PCR analysis of *PAK2*, *c-Myc* and *PKM2* upon PAK2 depletion and c-Myc complementation (n=3). **(S4c)** **(top)** protein expression of PKM2 was analysed through immunostaining followed by fluorescence microscopy, scale bar: 125 µm, **(bottom)** quantification of fluorescence intensity of immunostained PKM2 was measured with ImageJ software suite (n=2). **(S4d)** Binding of c-Myc at PKM promoter region (upstream to 5’ UTR) analyzed using Genomatix software suite. **(S4e)** The qRT-PCR analysis of *GLUT1*, *LDHA*, *LDHB, ENOLASE 1* in PAK2 depleted cells complemented with c-Myc expression (n=3). **(S4f)** Density of colonies were analyzed by staining the cells with crystal violet; **(left)** scanned image of crystal violet stained plate, **(right)** pixel density quantification of scanned plate with ImageJ software. Error bars shows mean values ± SD. Differences were considered statistically significant with a **p-value<0.01 and ***p-value<0.001.

**Supplementary Figure S5:** Global effect of PAK2 depletion **(S5a)** The qRT-PCR analysis of differentially expressed genes, Cyclin A2 (*CCNA2*), DNA Topoisomerase 2 Alpha (*TOP2A*), Polo Like Kinase 1 (*PLK1*), Proliferating Cell Nuclear Antigen (*PCNA*), Cyclin B2 (*CCNB2*), Cyclin B1(*CCNB1*), Cerebellar Degeneration Related Protein 1 (*CDR1*), Phospholipase A2 Group IIA (*PLA2G2A*), Defensin Beta 1 (*DEFB1*), Cluster Determinant 36 (*CD36*), Keratin 9 (*KRT9*), Endothelin 1 (*EDN1*) upon PAK2 depletion in H157 cancer cells (n=3). **(S5b)** The KEGG pathways analysis of proliferation pathway upon PAK2 depletion, analyzed using Transcriptome Analysis Console (TAC). **(S5c)** Gene ontology analysis of genes showed upregulation upon PAK2 depletion. Error bars shows mean values ± SD. Differences were considered statistically significant with a *p-value<0.05, **p-value<0.01 and ***p-value<0.001, ns, non-significant difference (p-value>0.05).
