# Supplementary material for: PAK2–c-Myc–PKM2 axis plays an essential role in head and neck oncogenesis via regulating Warburg effect
Source: Cell Death Dis. 2018 Aug 1;9(8):825. doi: 10.1038/s41419-018-0887-0 (PMC6070504; doi:10.1038/s41419-018-0887-0)
Supplement: Supplementary file 7 — Supplementary Table S1 [file 41419_2018_887_MOESM7_ESM.docx]

**SI Appendix Tables**

**Supplementary Table S1**: Clinicopathological characteristics of head and neck cancer patients.

| Sample ID | Gender | Age of Patient | Site of Cancer | Histopathology |
| --- | --- | --- | --- | --- |
| S-1 | Female | 70 | Tongue | Invasive Keratinizing SCC, G-I |
| S-2 | Male | 36 | Lower Lip | Invasive Keratinizing SCC, G-I |
| S-3 | Male | 55 | Tongue | Verrucous Carcinoma |
| S-4 | Male | 38 | Tongue | Invasive Keratinizing SCC, G-I |
| S-5 | Male | 32 | Buccal Mucosa | Keratinizing SCC, G-II |
| S-6 | Male | 40 | Retromolar Trigone | Well differentiated keratinizing SCC, G-I |
| S-7 | Male | 51 | Buccal Mucosa | Invasive Keratinizing SCC, G-II |
| S-8 | Male | 38 | Buccal Mucosa | Invasive Keratinizing SCC, G-III |
| S-9 | Female | 37 | Buccal Mucosa | Keratinizing SCC, G-I |
| S-10 | Male | 30 | Tongue | Invasive Keratinizing SCC, G-III |
| S-11 | Female | 45 | Alveolus | Keratinizing SCC, G-II |
| S-12 | Male | 32 | Buccal Mucosa | Invasive well differentiated Keratinizing SCC, G-I |
| S-13 | Male | 43 | Buccal Mucosa | Invasive Keratinizing SCC, G-I |
| S-14 | Male | 45 | Alveolus | Invasive Keratinizing SCC, G-I |
| S-15 | Male | 39 | Buccal Mucosa | Invasive Keratinizing SCC, G-II |
| S-16 | Male | 27 | GB Sulcus Maxilla | Keratinizing SCC, G-II |
| S-17 | Male | 61 | Buccal Mucosa | Verrucous Carcinoma |
| S-18 | Female | 47 | Left Buccal Mucosa | Grade-I |
| S-19 | Female | 55 | Retro Molar Trigone | Keratinizing SCC, Grade-III |
| S-20 | Female | 65 | Retro Molar Trigone | Invasive SCC, G-II |
| S-21 | Male | 70 | Alveolus | Early Invasive, well differentiated SCC, G-I |
| S-22 | Male | 40 | Buccal Mucosa | Invasive Keratinizing SCC, G-I |
| S-23 | Male | 39 | Buccal Mucosa | Invasive Keratinizing SCC, G-I |
| S-24 | Male | 60 | Tongue | Invasive mod. differentiated Keratinizing SCC, G-II |
| S-25 | Female | 55 | Tongue | Keratinizing SCC, G-II |
| S-26 | Male | 52 | Buccal Mucosa | Keratinizing SCC, G-I |
