# Supplementary material for: PAK2–c-Myc–PKM2 axis plays an essential role in head and neck oncogenesis via regulating Warburg effect
Source: Cell Death Dis. 2018 Aug 1;9(8):825. doi: 10.1038/s41419-018-0887-0 (PMC6070504; doi:10.1038/s41419-018-0887-0)
Supplement: Supplementary file 8 — Supplementary Table S2 [file 41419_2018_887_MOESM8_ESM.docx]

**SI Appendix Tables**

**Supplementary Table S2:** List of primer with sequences

Quantitative real-time PCR primers (q-RTPCR)

| Serial No. | Gene ID | 5’-oligo Sequence-3’ |
| --- | --- | --- |
| 1. | PAK2 F | GACTGCTCCTCCCGTTATTG |
| 2. | PAK2 R | ACTTGGCAGCACCATCAA |
| 3. | KDM1A F | TGTGAAGTGATAGCTGTGAATA |
| 4. | KDM1A R | CAAGAGCGAGCGGAAGAAG |
| 5. | DAPK3 F | CACGACATCTTCGAGAACAAGA |
| 6. | DAPK3 R | GCTTAGAGTGCAGGTAGTGAAC |
| 7. | MKi67 F | AGAGTCAGGTTCAGAAATCC |
| 8. | MKi67 R | TCTTTCTCCCTCCTCTCTT |
| 9. | CCND1 F | GTCTGCGAGGAACAGAAG |
| 10. | CCND1 R | GATGGAGTTGTCGGTGTAG |
| 11. | c-Myc F | TCCACCTCCAGCTTGTA |
| 12. | c-Myc R | TCGAGGAGAGCAGAGAAT |
| 13. | PKM2 F | TAGATTGCCCGTGAGGCAGAGGCT |
| 14. | PKM2 R | TGCCAGACTTGGTGAGGACGATTA |
| 15. | SAA1 F | TGGAGAGCCTACTCTGACAT |
| 16. | SAA1_R | CTGGATATTCTCTCTGGCATCG |
| 17. | BIRC3 F | CCGTGGAAATGGGCTTTAGTA |
| 18. | BIRC3_R | CCTCAGTTGCTCTTTCTCTCTC |
| 19. | FAM72D F | TTGCTGGCTGATACTGAAATAGA |
| 20. | FAM72D R | CAGGAAAGAAGACAGGAACTACA |
| 21. | PLK1_F | TCATCCAGAAGATGCTTCAGAC |
| 22. | PLK1 R | TGCTGGGAGCAATCGAAA |
| 23. | TOP2A_F | CAACAAACAAAGGGACCCAAA |
| 24. | TOP2A R | CTAGAAGTTAGGAGCTGTCCAAATA |
| 25. | ALAN F | ATTGAAGAACGAAGGACCTCAG |
| 26. | ALAN_R | TGGTTTCTGAACCGTACTGC |
| 27. | PCNA F | GCACTCAAGGACCTCATCAA |
| 28. | PCNA_R | TAGGTGTCGAAGCCCTCA |
| 29. | CCNB1 F | GGTGTCACTGCCATGTTTATTG |
| 30. | CCNB1 R | CGAAGGAAGTGCAAAGGTAGA |
| 31. | CCNB2_F | GGCTTCCAAGTATGAGGAGATG |
| 32. | CCNB2 R | CTCGCCTTAAGAAGTGTAGTGG |
| 33. | CCNA2_F | TCCTCCTTGGAAAGCAAACA |
| 34. | CCNA2 R | TTCTGGGTCCAGGTAAACTAATG |
| 35. | CDC20 F | CCACCATGATGTTCGGGTAG |
| 36. | CDC20_R | CCCTTGATGCTGGGTGAAT |
| 37. | CDK1 F | CCTATGGAGTTGTGTATAAGGGTAG |
| 38. | CDK1_R | GAGAAATTTCCCGAATTGCAGTA |
| 39. | AQP3 F | CCTCCCTTATCGTGTGTGTG |
| 40. | AQP3 R | CCCGCAAGGGCTGTAAA |
| 41. | CD36 F | GCTGTATTTGAATCCGACGTTAATC |
| 42. | CD36 R | CTTCTTTGCATTTGCTGATGTCT |
| 43. | TNFSF10 F | AGTCTCTCTGTGTGGCTGTA |
| 44. | TNFSF10 R | CTAACGAGCTGACGGAGTTG |
| 45. | KRT9 F | GAGCTTGGAAGACACGAAGA |
| 46. | KRT9 R | GGTTGTGGTAGGTCTCGATTT |
| 47. | DEFB1 F | CCTACCTTCTGCTGTTTACTCTC |
| 48. | DEFB1 R | CTTGGCCTTCCCTCTGTAAC |
| 49. | KRT1 F | CTCTGCTGGGATCATCAACTAC |
| 50. | KRT R | ACCTCCTCTAGCCACACTTAT |
| 51. | LDHA F | CGAAGACAAATTGAAGGGAGAGA |
| 52. | LDHA R | CGTGATAATGACCAGCTTGGA |
| 53. | LDHB F | TCTGTGACTGCCAATTCTAAGAT |
| 54. | LDHB R | CAGGACTGTACTTACTTGACGATCTG |
| 55. | GLUT1 F | CTCATGGGCTTCTCGAAACT |
| 56. | GLUT1 R | GTGACACTTCACCCACATACA |
| 57. | ENO1 F | TGTCATCAATGGCGGTTCTC |
| 58. | ENO1 R | GTGGTAAACCTCTGCTCCAAT |

c-Myc Chromatin Immunoprecipitation primers

| Serial No. | Oligo ID | 5’-oligo Sequence-3’ |
| --- | --- | --- |
| 1. | MYC_ChIP_1F | GACTTAAACACGACAAAGGTGAAA |
| 2. | MYC_ChIP_1R | GGAATCCTGCAGAGCGAAT |
| 3. | MYC_ChIP_2F | CTTCCTCCTGAAGGTGACTG |
| 4. | MYC_ChIP_2R | GCCTCAGCCTCAAGGTTAT |

Cloning primers

| Serial No. | Oligo ID | 5’-oligo Sequence-3’ |
| --- | --- | --- |
| 1. | c-Myc F | ATATGGATCCATGCCCCTCAACGTTAGCTTCAC |
| 2. | c-Myc R | ATATGAATTCTTACGCACAAGAGTTCCGTAGCT |
| 3. | PAK2_F | ATGCGGATCCATGTCTGATAACGGAGAACTGG |
| 4. | PAK2 R | ATGCCTCGAGTTAACGGTTACTCTTCATTGCTTC |

|  |
| --- |
|  |
